# Comparing Bayesian hierarchical meta-regression methods and evaluating the influence of priors for evaluations of surrogate endpoints on heterogeneous collections of clinical trials

**DOI:** 10.1186/s12874-024-02170-0

**Published:** 2024-02-16

**Authors:** Willem Collier, Benjamin Haaland, Lesley A. Inker, Hiddo J.L. Heerspink, Tom Greene

**Affiliations:** 1 Department of Population and Public Health Sciences, Keck School of Medicine, University of Southern California, Los Angeles, CA USA; 2 Department Population Health Sciences, University of Utah School of Medicine, Salt Lake City, UT USA; 3 Pentara Corporation, Millcreek, UT USA; 4 Division of Nephrology, Tufts University Medical Center, Boston, MA USA; 5 Department of Clinical Pharmacy and Pharmacology, Department of Nephrology, University of Groningen, Groningen, Netherlands

**Keywords:** Surrogate endpoint, Meta-regression, Bayesian hierarchical modeling, Chronic kidney disease

## Abstract

**Background:**

Surrogate endpoints, such as those of interest in chronic kidney disease (CKD), are often evaluated using Bayesian meta-regression. Trials used for the analysis can evaluate a variety of interventions for different sub-classifications of disease, which can introduce two additional goals in the analysis. The first is to infer the quality of the surrogate within specific trial subgroups defined by disease or intervention classes. The second is to generate more targeted subgroup-specific predictions of treatment effects on the clinical endpoint.

**Methods:**

Using real data from a collection of CKD trials and a simulation study, we contrasted surrogate endpoint evaluations under different hierarchical Bayesian approaches. Each approach we considered induces different assumptions regarding the relatedness (exchangeability) of trials within and between subgroups. These include partial-pooling approaches, which allow subgroup-specific meta-regressions and, yet, facilitate data adaptive information sharing across subgroups to potentially improve inferential precision. Because partial-pooling models come with additional parameters relative to a standard approach assuming one meta-regression for the entire set of studies, we performed analyses to understand the impact of the parameterization and priors with the overall goals of comparing precision in estimates of subgroup-specific meta-regression parameters and predictive performance.

**Results:**

In the analyses considered, partial-pooling approaches to surrogate endpoint evaluation improved accuracy of estimation of subgroup-specific meta-regression parameters relative to fitting separate models within subgroups. A random rather than fixed effects approach led to reduced bias in estimation of meta-regression parameters and in prediction in subgroups where the surrogate was strong. Finally, we found that subgroup-specific meta-regression posteriors were robust to use of constrained priors under the partial-pooling approach, and that use of constrained priors could facilitate more precise prediction for clinical effects in trials of a subgroup not available for the initial surrogacy evaluation.

**Conclusion:**

Partial-pooling modeling strategies should be considered for surrogate endpoint evaluation on collections of heterogeneous studies. Fitting these models comes with additional complexity related to choosing priors. Constrained priors should be considered when using partial-pooling models when the goal is to predict the treatment effect on the clinical endpoint.

**Supplementary Information:**

The online version contains supplementary material available at 10.1186/s12874-024-02170-0.

## Background

There is broad interest in the use of validated surrogate endpoints to expedite clinical trials in areas of slowly progressing disease, such as chronic kidney disease (CKD) [1–5]. A surrogate endpoint is typically a measure of disease progression captured earlier than an established clinical endpoint and should have the property that the treatment effect on the surrogate accurately predicts the treatment effect on the clinical endpoint [6–8]. This predictive potential is commonly established in a meta-regression analysis of previously conducted trials, where the meta-regression quantifies the strength of the association between treatment effects on the clinical and surrogate endpoints [3–8]. Accurate estimation of the meta-regression parameters requires variability in the treatment effects on the surrogate and clinical endpoints across trials used for analysis. To achieve this, the collection of trials can contain heterogeneity in terms of interventions and sub-classifications of disease [3, 4]. There is often interest among entities such as regulatory agencies regarding the performance of the surrogate in pre-specified, clinically or biologically motivated, and mutually exclusive subgroups defined by intervention or disease classes [1]. These interests introduce two specific goals the analytical approach must facilitate: The first is accurate estimation of subgroup-specific meta-regression parameters. The second is accurate prediction of treatment effects on the clinical endpoint, either for subgroups used in model fitting or for those not available for model fitting (e.g., for a novel intervention).

One meta-regression methodology involves a Bayesian hierarchical model, which can be used to account for estimation error of the treatment effects on both endpoints as well as the correlation of the sampling errors (a frequently used weighted generalized linear regression approach accounts only for sampling error of the effect estimate on one of the two endpoints) [6, 8, 9]. Under the hierarchical Bayesian approach, it is common to assume all trials used in the analysis to be fully exchangeable despite underlying differences in interventions or diseases across trials [4–6, 8]. In effect, this is accomplished by fitting a model with a single meta-regression relating treatment effects on the clinical endpoint to those of the surrogate endpoint to all trials available for the analysis, which we refer to as the “full-pooling” approach. Alternatively, distinct meta-regressions can be fit within subgroups in what we will refer to as the “no-pooling” approach [4, 7]. There are often too few trials and insufficient variability in treatment effects within subgroups to estimate the meta-regression parameters with satisfactory precision under a strict no-pooling strategy. An additional limitation to the full and no-pooling strategies is that each induces limitations to model-based prediction of the treatment effect on the clinical endpoint in a future trial. This is especially the case when there is interest in prediction for a trial which is of a “new subgroup”, one that was not available for the initial surrogacy evaluation. Afterall, in the ideal scenario a surrogate can be used for a trial evaluating a novel intervention or when applying an approved indication in a new patient population. Use of a full-pooling model requires the assumption that any future trial is fully exchangeable with the previous trials. Use of a no-pooling approach requires the future trial to be of a subgroup used for the surrogacy evaluation (“existing subgroup”).

Bayesian hierarchical meta-regression lends naturally to a “partial-pooling” compromise to these earlier approaches, where a between subgroup distribution is assumed for some or all subgroup-specific model parameters [7]. The partial-pooling approach relaxes the assumption of full-exchangeability of all trials used for the analysis, can improve precision of inference on subgroup-specific parameters due to data adaptive information sharing across subgroups, and provides a framework for model-based prediction of an effect on a clinical endpoint for a trial of either an existing or a new subgroup. However, critical decisions needed to fit models of this class are without empirical guidance in the literature. For example, use of fixed and random effects approaches are used interchangeably when employing full-pooling models, and the implications of these two approaches are not well understood under a partial-pooling model [8]. To our knowledge, there is also not yet work evaluating the impact of the choice of priors under partial-pooling strategies, even though the role of certain prior distributions is likely to be amplified in likely scenarios in which the number of subgroups is small.

In this paper, we provide results from a series of analyses intended to help guide practical decision making for surrogate endpoint evaluations on collections of heterogeneous studies. We explore the extent to which partial-pooling approaches improve precision in key posteriors of interest in surrogate evaluation, the extent to which bias occurs, contrast fixed and random effects variants of models described, and explore the impact of priors. In the Methods section, we describe the modeling approaches evaluated, priors, and how these methods can be used for prediction. In the Results section, we provide results of a limited simulation study and of an applied analysis of CKD trials. We then conclude with the Discussion section.

## Methods

### Modeling approaches to the trial-level analysis of a surrogate

For the trial-level evaluation of a surrogate endpoint, a two stage approach to the analysis is often used [6–8]. In the first stage, treatment effects on both the clinical and surrogate endpoint as well as standard errors and a within-study correlation between the error of the estimated effects are calculated for each trial. These trial-level measures are used as the data input in the meta-regression evaluation (the second stage). A two-level hierarchical model for the meta-regression can be used to account for within-study estimation error for both treatment effects [4–8].

Under the two-stage approach, one key distinction between commonly used second-stage models involves whether true treatment effects on the surrogate endpoint are viewed as fixed or random [6, 8]. Under the fixed effects approach, the true treatment effects on the surrogate endpoint are fixed and the true effects on the clinical endpoint are regressed on the true effects on the surrogate assuming Gaussian residuals. Under the random effects approach, the true treatment effects on both the surrogate and the clinical endpoints are assumed to follow a bivariate normal distribution [4, 5, 8]. The within-study joint distribution can be reasonably approximated with a bivariate normal distribution due to asymptotic normality, but the bivariate normality assumption for the between-study model is made for modeling convenience. Bujkiewicz et al. contrast the predictive performance of a surrogate under fixed and random effects approaches when using the full-pooling approach, but do not summarize differences in estimates of key parameters such as the meta-regression slope [8]. Papanikos et al. evaluate and contrast different fixed effects approaches in subgroup analyses of a surrogate, but do not compare fixed and random effects approaches [7]. We hypothesized that the fixed and random effects approaches could produce differing results because there may be more or less shrinkage in the true effects on the surrogate across trials (the “x-axis” variable in the regression) depending on the method used.

We next introduce the full pooling random and fixed effects models, which are applicable when the clinical trials being analyzed can be regarded as exchangeable. Let there be *N* total clinical trials, each of which compares an active treatment to a control. For trials $j = 1, \dots , N$, $(\widehat{\theta }_{1j}, \widehat{\theta }_{2j})'$ jointly represents the suitably scaled within study estimates of treatment effects on the clinical and surrogate endpoints for trial *j*. The pair $(\theta _{1j}, \theta _{2j})'$ represents the latent joint true treatment effects on the clinical and surrogate endpoints in study *j*. We let $\Sigma _j$ denote a within study variance-covariance matrix for study *j* ($\Sigma _{j1,1} = SE(\widehat{\theta }_{1j})^2$ is the squared standard error of the estimated clinical effect, $\Sigma _{j2,2} = SE(\widehat{\theta }_{2j})^2$ the squared standard error of the estimated surrogate effect, $\widehat{r}_j$ is the estimated within trial correlation for study *j*, implying $\Sigma _{j1,2} = \Sigma _{j2,1} = \widehat{r}_j SE(\widehat{\theta }_{1j}) SE(\widehat{\theta }_{2j})$). When the standard errors and within study correlation are available, it is customary to consider all entries of $\Sigma _j$ fixed and known [6–8, 10, 11]. For the random effects model, $\mu _s$ represents a population average true treatment effect on the surrogate, and $\sigma _s^2$ the between trial variance in true effects on the surrogate. We parameterize the model such that $\alpha$ denotes the meta-regression intercept, $\beta$ the slope, and $\sigma _e$ the residual standard deviation. The following represents the full-pooling random effects model (FP-RE).

$$\begin{aligned} (\widehat{\theta }_{1j}, \widehat{\theta }_{2j})' \sim N((\theta _{1j}, \theta _{2j})', \Sigma _j), \\ \theta _{2j} \sim N(\mu _s, \sigma _s^2), \quad \text {and} \quad \theta _{1j} | \theta _{2j} \sim N(\alpha + \beta \theta _{2j}, \sigma _e^2). \end{aligned}$$

To fit a full-pooling fixed effects model (FP-FE), rather than assuming a Gaussian distribution for which parameters will be estimated for $\theta _{2j}$ as above, an independent prior is assigned directly to each $\theta _{2j}$.

Next, suppose that the *N* trials are to be divided into *I* total subgroups because exchangeability is plausible for the trials within each subgroup but not necessarily between trials in different subgroups. In our experience, regulatory agencies have expressed concern of heterogeneity in surrogate quality across pre-specified subgroups present in the data being used to evaluate CKD-relevant surrogate endpoints. The models discussed throughout the remainder of this paper are thus intended for similar scenarios where: the *I* subgroups which motivate concern over the full exchangeability of trials (i.e., there might be a different association between treatment effects on the clinical and surrogate endpoint depending on the subgroup a trial pertains to) are presented to the statistical analyst independent of any statistical criteria, subgroup assignment for the trials available for model fitting is not ambiguous (e.g., the inclusion and exclusion criteria of a trial would easily determine the subgroup assignment if disease-based subgroups are of interest), and there can not be misclassification of trials into the wrong subgroups. When such an analytical scenario is presented, we might first consider fitting separate models within each subgroup. For $i = 1,\dots , I$, the following represents what we refer to as a no-pooling random effects (NP-RE) model for the $j^{\text {th}}$ trial within the $i^{\text {th}}$ subgroup.

$$\begin{aligned} (\widehat{\theta }_{1ji}, \widehat{\theta }_{2ji})' \sim N((\theta _{1ji}, \theta _{2ji})', \Sigma _{ji}), \\ \theta _{2ji} \sim N(\mu _{si}, \sigma _{si}^2), \quad \text {and} \quad \theta _{1ji} | \theta _{2ji} \sim N(\alpha _i + \beta _i \theta _{2ji}, \sigma _{ei}^2) \end{aligned}$$

We note that one could fit a no-pooling fixed-effects model by placing a prior directly on each $\theta _{2ji}$, rather than assuming the Gaussian distribution as above.

For the partial pooling approach, we can incorporate between-subgroup distributions as an intermediate layer in the Bayesian analysis to induce information sharing across subgroups [7, 12]. The terms controlling heterogeneity between subgroups are informed by the data. For example, if the data suggests a lack of between-subgroup heterogeneity for any given term, fitting this model should result in substantial information sharing and similar subgroup-specific parameter estimates. The partial pooling model may generate some amount of bias, but could counter-balance this bias with increased precision due to information sharing [12]. Among other reasons, because between-subgroup variation drives the data-adaptive information sharing, between-subgroup variance terms were of primary interest in our investigation of the influence of priors.

A partial-pooling random effects (PP-RE) model is displayed below. Consider there are additional model parameters necessary to define this model. We let $\mu _s$ and $\sigma _s^2$ represent the between subgroup average and variance of true treatment effects on the surrogate; $\alpha$ and $\sigma _{\alpha }^2$ and $\beta$ and $\sigma _{\beta }^2$ represent the between subgroup average and variance of the meta-regression intercept and slope, respectively; $\tau _s$ and $\tau _e$ denote the between-subgroup mean log-transformed true surrogate effects standard deviation and meta-regression residual standard deviation, respectively; $\gamma _s^2$ and $\gamma _e^2$ denote the between subgroup variance of the log-transformed within-subgroup true surrogate treatment effects standard deviation and meta-regression residual standard deviation, respectively.

1 $$\begin{aligned} (\widehat{\theta }_{1ji}, \widehat{\theta }_{2ji})' \sim N((\theta _{1ji}, \theta _{2ji})', \Sigma _{ji}),\end{aligned}$$

2 $$\begin{aligned} \theta _{2ji} \sim N(\mu _{si}, \sigma _{si}^2), \quad \theta _{1ji} | \theta _{2ji} \sim N(\alpha _i + \beta _i \theta _{2ji}, \sigma _{ei}^2),\end{aligned}$$

3 $$\begin{aligned} \mu _{si} \sim N(\mu _s, \sigma _s^2), \quad \alpha _i \sim N(\alpha , \sigma _{\alpha }^2), \quad \beta _i \sim N(\beta , \sigma _{\beta }^2)\end{aligned}$$

4 $$\begin{aligned} log(\sigma _{si}) \sim N(\tau _s, \gamma _s^2), \quad log(\sigma _{ei}) \sim N(\tau _e, \gamma _e^2). \end{aligned}$$

If fitting a partial-pooling fixed effects (PP-FE) model, a prior can be placed directly on each $\theta _{2ji}$, rather than assuming the hierarchical Gaussian distribution displayed above. We display an example of a PP-FE model here to contrast it with the PP-RE model more clearly. In this example, we place a N(0,10^2^) prior on each trial’s true treatment effect on the surrogate.

5 $$\begin{aligned} (\widehat{\theta }_{1ji}, \widehat{\theta }_{2ji})' \sim N((\theta _{1ji}, \theta _{2ji})', \Sigma _{ji}),\end{aligned}$$

6 $$\begin{aligned} \theta _{2ji} \sim N(0, 10^2), \quad \theta _{1ji} | \theta _{2ji} \sim N(\alpha _i + \beta _i \theta _{2ji}, \sigma _{ei}^2),\end{aligned}$$

7 $$\begin{aligned} \alpha _i \sim N(\alpha , \sigma _{\alpha }^2), \quad \beta _i \sim N(\beta , \sigma _{\beta }^2)\end{aligned}$$

8 $$\begin{aligned} log(\sigma _{ei}) \sim N(\tau _e, \gamma _e^2). \end{aligned}$$

To our knowledge, there has been just one other paper to evaluate partial-pooling strategies for the trial-level analysis of a surrogate. As discussed in the introduction, Papanikos et al. evaluated different fixed effects partial-pooling approaches [7]. An additional difference between the PP-FE model displayed above and those considered by Papaniko’s et al. is that there was not a between-subgroup distribution assumed for $\sigma _{ei}$ in their models. One advantage of allowing a between-subgroup distribution for $\sigma _{ei}$ is that it enables estimating posteriors for parameters defining between-subgroup distributions for all meta-regression parameters (intercept, slope, and residual variance). This subsequently facilitates prediction for a trial of a new subgroup, as is discussed in the Generating posterior predictive distributions section.

### Analysis set 1: simulation study

We generated trial level summary data (estimated treatment effects, standard errors, and the within-study correlations) based on four broad simulation setups, where within each we introduced two variants depending on the distribution used to simulate true treatment effects on the surrogate. The setups considered were motivated by applied data used to evaluate GFR slope. We consider three subgroups of trials as in previous evaluations of GFR slope and to reflect the likely scenarios where the available data limits the number of subgroups, stressing the potential for benefit from data adaptive partial-pooling [4]. We simulated 15 medium-to-large trials per subgroup (standard errors on either endpoint reflect trials with roughly 300-2000 patients). Within-study correlations were drawn equally at random from the range of values present in our application data. Without loss of generalizability, we modeled a negative trial-level association. As discussed in the section titled Analysis set 2: application analysis of CKD trials, there is a negative association between treatment effects on the clinical endpoint and treatment effects on GFR slope. We also varied the sizes of subgroups and the degree of between-study variability in true effects on the surrogate. Broadly, we consider one setup (S1) where there is homogeneity in the quality of the surrogate across subgroups, another setup (S2) where the surrogate is weak in two subgroups and strong in another, another setup (S3) where the surrogate is weak in one subgroup and strong in the other two, and a final setup (S4) where surrogate quality is different in all three subgroups. The strength of the surrogate was defined by the true meta-regression $R^2$. Earlier work has proposed that $R^2 \in (0,0.49)$, $R^2 \in (0.5,0.72)$, and $R^2 \in (0.73,1)$ suggest a weak, moderate, and strong surrogate, respectively [13]. For our purposes, we simulated data from true parameter values to obtain $R^2 = 0.35,0.65,0.95$ to define the surrogate as weak, moderate or strong within subgroups, respectively.

Consider the data generating model below for the first variant (V1) of the four simulation setups. To simulate estimated clinical and surrogate effects for trial *j* ($j = 1,\dots , 15$) in subgroup *i* ($i = 1,2,3$) when true surrogate effects are Gaussian, we first drew true surrogate effects from (9), then drew conditional true clinical effects from (10), and finally drew a pair of estimated effects using (11). The standard errors and within-study correlations forming the matrices $\Sigma _{ji}$ were drawn according to the rules described above using uniform distributions to reflect variation in trial sizes.

9 $$\begin{aligned} \theta _{2ji} \sim N(\mu _{si}, \sigma _{si}^2),\end{aligned}$$

10 $$\begin{aligned} \theta _{1ji} | \theta _{2ji} \sim N(\alpha _i + \beta _i \theta _{2ji}, \sigma _{ei}^2)\end{aligned}$$

11 $$\begin{aligned} (\widehat{\theta }_{1ji}, \widehat{\theta }_{2ji})' \sim N((\theta _{1ji}, \theta _{2ji})', \Sigma _{ji}) \end{aligned}$$

We also sought to contrast results under the different models when true treatment effects on the surrogate were distinctly non-Gaussian (V2). We used the following data generating model, where true effects on the surrogate for each trial were drawn from a bimodal distribution (12).

12 $$\begin{aligned} \theta _{2ji} \sim 0.5N(\mu _{1si}, \sigma _{si}^2) + 0.5N(\mu _{2si}, \sigma _{si}^2)\end{aligned}$$

13 $$\begin{aligned} \theta _{1ji} | \theta _{2ji} \sim N(\alpha _i + \beta _i \theta _{2ji}, \sigma _{ei}^2)\end{aligned}$$

14 $$\begin{aligned} (\widehat{\theta }_{1ji}, \widehat{\theta }_{2ji})' \sim N((\theta _{1ji}, \theta _{2ji})', \Sigma _{ji}) \end{aligned}$$

To summarize results, we provide simulation average posterior medians, $2.5^{\text {th}}$ and $97.5^{\text {th}}$ percentiles for models fit across 100 simulated datasets per setup. We also summarize posterior predictive distributions (PPDs - described further below).

### Analysis set 2: application analysis of CKD trials

We compare analyses using the models discussed above on a set of 66 CKD studies. Data from these studies was collected by the Chronic Kidney Disease Epidemiology Collaboration (CKD-EPI), an international research consortium [3, 4]. Evaluations of GFR slope on this collection of studies have been described extensively [3, 4]. For the purposes of this paper, we focus on the GFR “chronic slope” as the surrogate [4]. Time-to-doubling of serum creatinine or kidney failure is used as the clinical endpoint, which is accepted by regulatory agencies and is widely used as the primary endpoint in pivotal phase 3 clinical trials of CKD [3]. Treatment effects on the clinical endpoint were expressed as log transformed hazard ratios (HRs), estimated using proportional hazards regression. A shared parameter mixed effects model was used to jointly model longitudinal GFR trajectories and the time of termination of GFR follow-up due to kidney failure or death for each randomized patient. Treatment effects on the chronic GFR slope are expressed as the mean difference in the treatment arm slope minus the control arm slope, expressed in ml/min/1.73 m^2^ per-year. Further detail on the methods used to estimate effects on GFR slope-based endpoints are described elsewhere in the literature [4, 14]. Finally, we obtained robust sandwich estimates of the within-study correlations using a joint model as in previous work by CKD-EPI [4].

Heterogeneity across the CKD-EPI trials can be attributed to many study level factors. We consider four disease-defined subgroups (CKD with unspecified cause (CKD-UC), diabetes (DM), glomerular diseases (GN), and cardiovascular diseases (CVD)) and 16 intervention-defined subgroups (listed in the Additional file 1: Section 1). For the application analyses, we focus on fitting the FP-RE and PP-RE models, and use different sets of priors under the PP-RE model (we also contrast results under the PP-RE and PP-FE models where subgroups are defined by disease to complement certain simulation analyses). To capture the scenario where there is interest in prediction for a future trial of a new subgroup, we first fit models by leaving out CVD studies, and we generated PPDs for those studies left-out. For intervention-defined subgroups, we fit the model for trials of 7 subgroups for which there were at least 3 studies, and we then generated PPDs for studies of the remaining left-out, smaller subgroups. We also summarize PPDs obtained for studies of the subgroups used for model fitting under these two subgroup schema.

### Priors

For the purposes of the simulation study, we utilized diffuse priors, which is a common practice in surrogate endpoint evaluations [4, 6–8]. For the full-pooling and no-pooling models, we used the $N(0,10^2)$ prior for the intercept ($\alpha$ or $\alpha _i$) and slope ($\beta$ or $\beta _i$), and for the mean true treatment effect on the surrogate ($\mu _s$, $\mu _{si}$ under random effects models) or for trial-specific true effects on the surrogate when fitting the fixed effects models ($\theta _{2ji}$). As in previous work in CKD, we used inverse-gamma priors on variance terms ($\text {IG(a,b)}$ for shape $\text {a}$ and scale $\text {b}$) [4, 5]. For the full-pooling and no-pooling models, we used $\sigma _{ei}^2,\sigma _{e}^2 \sim \text {IG}(0.001,0.001)$. Where appropriate (random effects models), we also used $\sigma _s^2,\sigma _{si}^2 \sim$ $\text {IG}(0.001,0.001)$. The $\text {IG}(0.001,0.001)$ prior is considered an approximation to the Jeffery’s prior. For partial-pooling models, we let $\tau _e^2 \sim \text {IG}(0.0025,0.001)$ and $\gamma _e \sim \text {half-normal}(0,3^{2})$, and for the random effects variants $\tau _s^2 \sim \text {IG}(0.0025,0.001)$ and $\gamma _s \sim \text {half-normal}(0,3^2)$. This combination translates to priors for within subgroup standard deviations in the partial-pooling models matching those of the no-pooling models to the extent that the 25^th^, 50^th^, and 75^th^ prior percentiles differed by less than 0.05. For $\sigma _{\alpha }$, $\sigma _{\beta }$ , $\sigma _{s}$, we used $\text {half-normal}(0,2^2)$. These specific half-normal priors should be considered highly diffuse for all of our analyses.

For our application analyses, we considered three variations on priors when employing the PP-RE model. We considered different priors for partial-pooling models because we hypothesized that not only narrow priors, but also highly diffuse priors could unduly influence certain results of our analyses. This is because there is often a limited number of studies available for meta-analysis, which can limit the number of subgroups. The categorization of studies based on constructs such as disease subtype or treatment comparison class may also provide a small number of subgroups. When there are just a few subgroups, the data provides very little information on subgroup-to-subgroup variation. The posteriors for between-subgroup variance terms may be more likely to exhibit minimal updates from the priors based on the data. As such, if priors are so diffuse that they represent a range of variability that is beyond practical reality, so too could the posteriors. As described below, this is also important because between-subgroup variance parameters are utilized in generating posterior predictive distributions for a trial of a new subgroup. A practical degree of narrowing certain priors could be seen as a necessary middle ground between use of overly narrow or overly diffuse priors. While we narrowed all priors for our constrained “sets” considered, the priors we focused on were for between-subgroup standard deviations for meta-regression parameters. We first used the fully diffuse priors displayed above. We then employed an iterative procedure, where we narrowed priors (emphasizing between-subgroup standard deviation parameters such as $\sigma _{\alpha },\sigma _{\beta },\gamma _e$) until a set was found that produced no more than 0.05 difference in the posterior median, 2.5^th^, and 97.5^th^ percentiles for the within-subgroup meta-regression posteriors, no matter how much narrower posteriors on between-subgroup parameters became (referred to as “Constrained Priors Set 1”, which were ultimately the same for either subgroup classification). Finally, we chose what we will refer to as “domain-constrained” priors (“Constrained Priors Set 2”). It is reasonable to choose a prior that constrains between-subgroup variability to a range that is actually plausible in reality based on subject matter expertise (e.g., through a prior elicitation process). For example, in our case the intercept is the expected true log-HR on the clinical endpoint when the true effect on the surrogate is the null effect. When there is a null-effect on the surrogate, we may suspect a low probability of an expected HR on the clinical endpoint that is very strong in either direction (e.g., below 0.5 or above 2.0), and this logic can be used to provide a moderate to low probability for subgroup-specific intercepts to go beyond these values. Domain-constrained priors were the narrowest among those considered for our analyses, and further detail on choosing these priors is provided in Section 2 of Additional file 1.

We wish to also emphasize that there is an important distinction between narrowing priors for the terms that define variability in the treatment effects on the surrogate across studies, and for the meta-regression parameters. The degree of variability of treatment effects on the surrogate influences the extent to which the data allows the quality of the surrogate to be inferred. Priors for the distribution(s) of true treatment effects on the surrogate should be left sufficiently diffuse so as not to restrict variation in effects across studies. In our cases, these were narrowed because the diffuse priors typically used are excessively wide relative to the range of treatment effects that are reasonable. The priors of primary interest are again those governing the degree of variability between subgroups in the meta-regression terms (e.g., $\sigma _{\beta }$).

### Generating posterior predictive distributions

There are a number of strategies that can be used to generate PPDs for the treatment effect on the clinical endpoint based on the treatment effect on the surrogate. In our simulation study, we compare summaries of PPDs for the true treatment effect on the clinical endpoint, which only takes into account uncertainty in the estimated meta-regression parameters. This is possible in a simulation analysis because we actually know the true effect on the surrogate [7]. For each study left-out of model fitting, let the true effect on the surrogate for that study be denoted $\theta _{2}^N$. Then, the PPD for the true effect on the clinical endpoint is generated by taking $m=1,\dots ,M$ draws (for each of *M* posterior draws obtained in model fitting) from $N(\alpha ^{*m} + \beta ^{*m}\theta _{2}^N,\sigma _e^{*m2})$, where $\alpha ^{*m}, \beta ^{*m}, \sigma _e^{*m}$ represent draws from posteriors from either the full-pooling, no-pooling or partial-pooling models. For our purposes, subgroup-specific parameters were used when trials were simulated from the same subgroup if using no-pooling or partial-pooling.

In application analyses, it is only possible to obtain the PPD for the estimated effect on the clinical endpoint, which involves a procedure that takes into account not only uncertainty in the meta-regression posteriors, but also uncertainty due to sampling error in the treatment effect estimates. Section 3 of the Additional file 1 provides further detail on the procedures used for prediction in our application analyses. We provide an overview here. For one part of our application analyses, we generated PPDs for trials of existing subgroups. Under full-pooling models, we directly used the single set of estimated meta-regression posteriors to map the effect on the surrogate to a predicted effect on the clinical endpoint. Under no-pooling and partial-pooling models, we used the appropriate subgroup-specific meta-regression posteriors estimated directly in model fitting (e.g., to make a prediction for a trial of subgroup $i \in \{1,\dots ,I\}$ we directly use a draw from the posterior for $\beta _i$ obtained through model fitting). In our second prediction exercise we generated PPDs for trials of a new subgroup. Only the full-pooling and partial-pooling models were used as no-pooling models do not facilitate estimation of parameters which allow the surrogate to be applied in a new subgroup. Again, under full-pooling models we used the single set of estimated meta-regression posteriors, which induces the assumption that the new study is fully exchangeable with those used for model fitting despite that it pertains to a new subgroup. Under partial-pooling models we used draws from population subgroup distributions (e.g., we draw a new $\beta _{\text {new}}$ from $N(\beta ,\sigma _{\beta }^2)$) to map the effect on the surrogate to the predicted clinical effect (that this process requires $\sigma _{\beta }$, which again may be influenced by the choice of priors in practical scenarios where the number of subgroups is small, is what motivated our interest in careful choosing of priors). This way, for all prediction exercises we were using subgroup-specific meta-regression posteriors for prediction, just that these were random draws from the population distribution when applying the surrogate to a new setting under the partial-pooling approach. When we are extrapolating the trial-level association to a new subgroup, drawing from the population distribution for each meta-regression posterior induces an additional degree of uncertainty into the prediction. This could be seen as a reasonable compromise between applying the fitted full-pooling model, which ignores that the new study represents a new scenario, and not applying the surrogate at all (i.e., the no-pooling approach). As discussed when introducing the PP-RE approach, the reason why we assume between-subgroup distributions for $\sigma _e$ is to facilitate the possibility of drawing subgroup-specific residual standard deviations needed in prediction for a trial of a new subgroup.

### Software

For simulation and applied analyses, we used the University of Utah Center for High Performance Computing Linux cluster. On the cluster, we used R version 4.0.3 for data preparation and for generating model summaries. The mcmc sampling algorithms for model fitting were implemented using RStan version 2.21.12 [15]. We utilized the Gelman-Rubin statistic to assess adequate convergence of chains and the effective sample size to evaluate whether there were sufficient mcmc draws to utilize certain posterior summaries such as tail percentiles (as well as additional visual summaries such as rank plots) [16, 17]. We landed on 10,000-20,000 mcmc iterations and 3 independent chains across all analyses. Finally, for the application analyses, the SAS NLMixed procedure was used to estimate treatment effects on the clinical and surrogate endpoints, standard errors, and within-study correlations within each study [18]. Example RStan code (PP-RE model) and R code (for simulating data) is provided in Section 4 of Additional file 1.

## Results

### Simulation study results

#### Contrasting different random effects approaches under gaussian surrogate effects

Table 1 provides summaries of posterior distributions obtained from fitting models on simulation setups 1-4 (V1 and V2). When there was no heterogeneity in the true meta-regression parameters across subgroups (Setup 1), the PP-RE model resulted in limited additional uncertainty in posteriors relative to the FP-RE model, and also resulted in negligible additional bias via the posterior medians. Across Setups 2-4, where the strength of the association between effects on the clinical and surrogate endpoint varied across subgroups, for any given meta-regression parameter summarized, use of the FP-RE model naturally obscured such heterogeneity. The NP-RE and PP-RE models more adequately produced subgroup-specific meta-regression posteriors that suggested heterogeneity in the quality of the surrogate, but in every case the PP-RE model produced more precise posteriors than that of the NP-RE model. Benefits were especially evident when focusing on posteriors for the meta-regression slope. While the PP-RE model typically resulted in a small degree of bias, between-subgroup heterogeneity was potentially more evident due to improved precision. Precision gains under the PP-RE over the NP-RE model were also observed in the sensitivity analyses considered (Tables 2 and 3 of Additional file 1), including where there was heterogeneity in subgroup sizes. There was a larger degree of pooling away from parameter values true for smaller subgroups under partial-pooling, but the PP-RE model still allowed for heterogeneity in posterior medians and 95% credible intervals to aid in understanding variations in surrogate quality across subgroups. One potential drawback of all approaches considered was that $R^2$ posterior medians appeared biased in every scenario evaluated, reflecting the challenge associated with accurate estimation of $R^2$ with limited data. The average posterior median $R^2$ under partial-pooling was more biased than under no-pooling in certain scenarios such as where the surrogate was weak, possibly due to information sharing. The challenges associated with estimating $R^2$ emphasize why it is important to consider not only reporting $R^2$ point estimates but also credible intervals. The credible intervals under the PP-RE approach remained wide in subgroups where the surrogate was weak. Differences in model performance were also evident in evaluations of model-based prediction of treatment effects on the clinical endpoint (Table 2). Coverage of true clinical effects by 95% posterior prediction intervals was lower when using the FP-RE model even where meta-regression parameters were truly the same across subgroups. The NP-RE model resulted in highest coverage because of excessively wide prediction intervals, whereas prediction under the PP-RE model resulted in improved precision with adequate coverage.

**Table 1:** Summary of analyses from simulation setups 1-4

Data Simulated:	Gaussian True Surrogate Effects			Non-Gaussian True Surrogate Effects	
	FP-RE Summary	NP-RE Summary	PP-RE Summary	PP-RE Summary	PP-FE Summary
Setup 1 (Truth)					
$\alpha _1$ (0)	0.00(-0.09,0.11)	0.02(-0.46,0.57)	-0.01(-0.14,0.13)	0.00(-0.12,0.13)	-0.05(-0.15,0.06)
$\alpha _2$ (0)		0.00(-0.45,0.49)	-0.01(-0.13,0.14)	0.00(-0.12,0.14)	-0.05(-0.15,0.06)
$\alpha _3$ (0)		0.01(-0.46,0.57)	-0.01(-0.131,0.13)	0.01(-0.12,0.15)	-0.05(-0.16,0.07)
$\beta _1$ (-0.45)	-0.45(-0.63,-0.31)	-0.49(-1.44,0.33)	-0.44(-0.67,-0.24)	-0.46(-0.68,-0.27)	-0.35(-0.49,-0.22)
$\beta _2$ (-0.45)		-0.46(-1.52,0.51)	-0.45(-0.68,-0.25)	-0.46(-0.69,-0.27)	-0.35(-0.50,-0.22)
$\beta _3$ (-0.45)		-0.47(-1.53,0.49)	-0.44(-0.65,-0.25)	-0.45(-0.67,-0.26)	-0.35(-0.49,-0.21)
$\sigma _{e1}$ (0.05)	0.06(0.02,0.14)	0.08(0.02,0.22)	0.06(0.01,0.17)	0.06(0.01,0.17)	0.07(0.01,0.18)
$\sigma _{e2}$ (0.05)		0.08(0.02,0.22)	0.06(0.01,0.17)	0.07(0.01,0.17)	0.07(0.01,0.18)
$\sigma _{e3}$ (0.05)		0.08(0.02,0.21)	0.06(0.01,0.16)	0.06(0.01,0.17)	0.07(0.01,0.18)
$R^2$ 1 (0.95)	0.90(0.60,0.99)	0.80(0.20,0.99)	0.89(0.41,1.00)	0.91(0.49,1.00)	0.92(0.57,1.00)
$R^2$ 2 (0.95)		0.78(0.23,0.98)	0.88(0.40,1.00)	0.90(0.48,1.00)	0.91(0.55,1.00)
$R^2$ 3 (0.95)		0.80(0.23,0.98)	0.90(0.46,1.00)	0.90(0.48,1.00)	0.92(0.56,1.00)
Setup 2 (Truth)					
$\alpha _1$ (0)	0.00(-0.11,0.13)	-0.01(-0.43,0.39)	0.01(-0.12,0.15)	0.01(-0.11,0.14)	-0.02(-0.12,0.09)
$\alpha _2$ (0)		0.01(-0.57,0.62)	0.01(-0.14,0.18)	0.01(-0.12,0.16)	-0.03(-0.14,0.09)
$\alpha _3$ (0)		0.03(-0.54,0.77)	-0.02(-0.20,0.18)	-0.02(-0.20,0.17)	-0.09(-0.25,0.05)
$\beta _1$ (-0.25)	0.47(-0.70,-0.27)	-0.25(-1.93,1.54)	-0.32(-0.73,0.06)	-0.33(-0.66,0.00)	-0.24(-0.42,-0.05)
$\beta _2$ (-0.35)		-0.40(-1.85,0.97)	-0.40(-0.75,-0.09)	-0.40(-0.72,-0.11)	-0.28(-0.46,-0.11)
$\beta _3$ (-0.6)		-0.65(-1.88,0.29)	-0.57(-0.88,-0.30)	-0.55(-0.85,-0.30)	-0.42(-0.61,-0.24)
$\sigma _{e1}$ (0.15)	0.13(0.06,0.22)	0.11(0.03,0.27)	0.10(0.02,0.23)	0.11(0.03,0.23)	0.11(0.03,0.24)
$\sigma _{e2}$ (0.115)		0.09(0.03,0.24)	0.09(0.02,0.20)	0.10(0.02,0.22)	0.10(0.03,0.22)
$\sigma _{e3}$ (0.06)		0.09(0.03,0.26)	0.08(0.01,0.21)	0.09(0.02,0.22)	0.10(0.02,0.22)
$R^2$ 1 (0.35)	0.69(0.33,0.91)	0.45(0.02,0.93)	0.53(0.07,0.95)	0.59(0.10,0.95)	0.66(0.16,0.95)
$R^2$ 1 (0.65)		0.61(0.07,0.96)	0.69(0.14,0.98)	0.71(0.17,0.97)	0.76(0.25,0.97)
$R^2$ 3 (0.95)		0.84(0.29,0.99)	0.85(0.38,1.00)	0.84(0.37,0.99)	0.87(0.48,0.99)
Setup 3 (Truth)					
$\alpha _1$ (0)	0.01(-0.10,0.13)	0.02(-0.39,0.43)	0.03(-0.10,0.18)	0.01(-0.11,0.14)	-0.02(-0.12,0.09)
$\alpha _2$ (0)		0.02(-0.47,0.62)	0.00(-0.15,0.17)	-0.01(-0.15,0.15)	-0.07(-0.19,0.05)
$\alpha _3$ (0)		0.05(-0.53,0.81)	0.00(-0.17,0.20)	-0.01(-0.18,0.18)	-0.09(-0.24,0.05)
$\beta _1$ (-0.25)	-0.56(-0.80,-0.36)	-0.33(-1.90,1.20)	-0.40(-0.80,-0.03)	-0.36(-0.71,-0.03)	-0.25(-0.44,-0.06)
$\beta _2$ (-0.6)		-0.65(-2.09,0.51)	-0.59(-0.95,-0.31)	-0.58(-0.91,-0.32)	-0.42(-0.61,-0.24)
$\beta _3$ (-0.6)		-0.68(-1.93,0.26)	-0.60(-0.93,-0.35)	-0.58(-0.87,-0.34)	-0.43(-0.61,-0.26)
$\sigma _{e1}$ (0.15)	0.11(0.05,0.20)	0.11(0.04,0.27)	0.10(0.02,0.23)	0.10(0.03,0.23)	0.11(0.03,0.24)
$\sigma _{e2}$ (0.06)		0.09(0.03,0.25)	0.08(0.01,0.20)	0.09(0.01,0.21)	0.10(0.02,0.23)
$\sigma _{e3}$ (0.06)		0.09(0.03,0.25)	0.08(0.01,0.20)	0.08(0.01,0.21)	0.09(0.02,0.22)
$R^2$ 1 (0.35)	0.80(0.46,0.95)	0.51(0.04,0.94)	0.64(0.11,0.96)	0.64(0.12,0.96)	0.69(0.18,0.95)
$R^2$ 1 (0.95)		0.81(0.20,0.99)	0.87(0.36,1.00)	0.88(0.41,1.00)	0.88(0.47,0.99)
$R^2$ 3 (0.95)		0.86(0.29,0.99)	0.89(0.42,1.00)	0.87(0.43,0.99)	0.89(0.51,0.99)
Setup 4 (Truth)					
$\alpha _1$ (0)	0.01(-0.11,0.13)	-0.01(-0.45,0.42)	0.01(-0.13,0.16)	0.01(-0.12,0.14)	-0.01(-0.12,0.10)
$\alpha _2$ (0)		0.00(-0.64,0.62)	0.01(-0.16,0.19)	0.02(-0.13,0.18)	-0.01(-0.13,0.11)
$\alpha _3$ (0)		0.02(-0.59,0.80)	-0.03(-0.23,0.18)	-0.03(-0.22,0.18)	-0.09(-0.25,0.06)
$\beta _1$ (-0.25)	-0.44(-0.68,-0.23)	-0.22(-1.91,1.50)	-0.31(-0.73,0.10	-0.31(-0.65,0.02)	-0.23(-0.41,-0.04)
$\beta _2$ (-0.25)		-0.26(-1.84,1.35)	-0.31(-0.70,0.06)	-0.33(-0.67,0.00)	-0.23(-0.42,-0.04)
$\beta _3$ (-0.6)		-0.64(-1.93,0.37)	-0.55(-0.89,-0.25)	-0.55(-0.85,-0.28)	-0.41(-0.61,-0.23)
$\sigma _{e1}$ (0.15)	0.15(0.08,0.24)	0.12(0.04,0.28)	0.11(0.03,0.23)	0.11(0.03,0.24)	0.12(0.04,0.24)
$\sigma _{e2}$ (0.15)		0.12(0.04,0.27)	0.11(0.03,0.24)	0.12(0.03,0.24)	0.12(0.04,0.25)
$\sigma _{e3}$ (0.06)		0.09(0.03,0.25)	0.09(0.02,0.22)	0.10(0.02,0.23)	0.11(0.02,0.23)
$R^2$ 1 (0.35)	0.60(0.24,0.87)	0.47(0.03,0.93)	0.50(0.06,0.94)	0.56(0.08,0.94)	0.63(0.14,0.94)
$R^2$ 1 (0.35)		0.47(0.03,0.94)	0.51(0.06,0.93)	0.55(0.07,0.94)	0.63(0.12,0.93)
$R^2$ 3 (0.95)		0.84(0.28,0.99)	0.82(0.31,0.99)	0.82(0.34,0.99)	0.86(0.44,0.99)

Summaries include the posterior median and, in parentheses, the 95% credible interval, averaged across simulations

**Table 2:** Posterior predictive comparisons on simulated data

	FP-RE	NP-RE	PP-RE				PP-RE	PP-FE		
	Cvg	Cvg	Cvg	RR_np_	WR_np_		Cvg	Cvg	RR_fe_	WR_fe_
Setup 1 (V1)						Setup 1 (V2)				
SG 1	0.99	1.00	1.00	1.813	2.685	SG 1	1.00	0.97	1.352	0.861
SG 2	0.99	1.00	1.00	1.549	3.027	SG 2	1.00	0.97	1.319	0.844
SG 3	0.99	1.00	1.00	1.773	3.246	SG 3	1.00	0.97	1.362	0.857
Setup 2 (V1)						Setup 2 (V2)				
SG 1	0.87	0.95	0.90	2.035	2.642	SG 1	0.90	0.90	0.912	1.041
SG 2	0.94	0.96	0.94	2.250	2.766	SG 2	0.94	0.93	0.937	1.078
SG 3	0.97	0.99	0.99	2.234	2.600	SG 3	0.99	0.91	1.584	1.057
Setup 3 (V1)						Setup 3 (V2)				
SG 1	0.79	0.95	0.89	1.892	2.492	SG 1	0.89	0.90	0.887	1.000
SG 2	1.00	1.00	0.99	1.772	2.668	SG 2	0.99	0.95	1.529	1.090
SG 3	0.99	0.99	0.99	1.925	2.726	SG 3	0.99	0.92	1.670	1.057
Setup 4 (V1)						Setup 4 (V2)				
SG 1	0.92	0.96	0.92	1.886	2.502	SG 1	0.92	0.91	0.923	1.116
SG 2	0.89	0.94	0.90	1.888	2.630	SG 2	0.90	0.90	0.881	1.046
SG 3	0.98	0.99	0.99	1.697	2.507	SG 3	0.99	0.92	1.592	1.112

*V1 *Setups where true surrogate effects are Gaussian, *V2 *Setups with Non-Gaussian true surrogate effects, *SG *“Subgroup.”, *Cvg* Coverage, *RR*_np_ Ratio of NP-RE prediction RMSE over PP-RE prediction RMSE, *WR*_np_: Ratio of NP-RE average 95% PPD width to PP-RE average 95% PPD width, *RR*_fe_ Ratio of PP-FE prediction RMSE over PP-RE prediction RMSE, *WR*_fe_ Ratio of PP-FE average 95% PPD width to PP-RE average 95% PPD width

#### Contrasting fixed vs. random effects partial-pooling models under non-Gaussian surrogate effects

Where the true treatment effects on the surrogate were non-Gaussian, the PP-FE model resulted in downward bias in meta-regression intercept posteriors (e.g., via the posterior median), whereas the PP-RE model either did not result in any bias or resulted in a lesser degree of bias. The PP-FE model also resulted in downward bias in the meta-regression slope posteriors (regression dilution bias) in subgroups where the surrogate was simulated to be moderate-to-strong. We hypothesize that this downward bias was due to the absence of shrinkage of true treatment effects on the surrogate (the “x-axis” variable in the meta-regression) towards one another. Because no common distribution is assumed for true effects on the surrogate across studies, the true effects are likely to be more dispersed in contrast to use of the random effects model, where the Gaussian distributional assumption could result in pooling of true treatment effects on the surrogate across studies. Although the random effects model resulted in a small degree of upward bias in the meta-regression slope in subgroups where the surrogate was weak, the $R^2$ posteriors were wider and their median’s lower than under the fixed effects model. This means that the risk of concluding a stronger surrogate than was true in reality was mitigated due to the less optimistic $R^2$ posteriors. The implications of these biases observed in meta-regression posteriors are also evidenced in summaries of prediction in Table 2. Despite the use of fixed effects, coverage of the true treatment effect on the clinical endpoint by 95% posterior predictive intervals under the PP-FE model was poorer than under the PP-RE model, to the largest extent in subgroups where the surrogate was strongest, which is likely where prediction is of greatest interest.

### Application analysis results

The primary goal of the application analysis was to compare meta-regression posteriors and PPDs obtained after fitting the PP-RE model with different priors. However, we also note that Fig. 7 in the Additional file 1 indicates differences in the meta-regression slope estimates under the PP-RE and PP-FE models from the analysis where models were fit to disease-defined subgroups. The discrepancy in the posterior median between the two models grew larger for subgroups with a stronger meta-regression slope under the PP-RE model (under the PP-RE model, medians were -0.25, -0.30, -0.35, whereas, under the PP-FE model, these were -0.27, -0.29, -0.29).

Table 3 summarizes meta-regression slope posteriors from the application analyses (3 disease-defined subgroups, with 59 studies for model fitting in one analysis and 7 intervention-defined subgroups with 51 studies used for model fitting in the other). Additional file 1: Tables 5 and 6 contain posterior summaries for the full set of meta-regression parameters from these analyses. When there were three disease-defined subgroups, using increasingly narrow priors resulted not only in narrower posteriors for between-subgroup standard deviation parameters but also for the between-subgroup mean parameters (even when priors for between-subgroup means were left the same). However, priors could be narrowed considerably before the within-subgroup posteriors narrowed. In most cases, even the narrowest priors used did not meaningfully change the inference on subgroup-specific posteriors. When there were 7 subgroups, narrower priors again resulted in equivalent or narrower posteriors for between-subgroup means and standard deviations, but to a lesser extent when compared to the analysis with fewer subgroups. Similarly, the use of narrower priors resulted in little, if any change in the within-subgroup posteriors under the options considered for intervention-defined subgroups.

**Table 3:** Application: results under the partial pooling random-effects model with different priors

Parameter (Subgroup, N Studies)	Diffuse Priors	Constrained Priors Set 1	Constrained Priors Set 2
Analysis: Cardiovascular Studies Left-Out (3 Subgroups in Model Fitting)			
Mean $\beta$	-0.30(-0.85,0.2)	-0.30(-0.68,0.02)	-0.30(-0.61,-0.06)
Between-Subgroup SD of $\beta$	0.15(0.01,1.53)	0.13(0.01,0.77)	0.12(0.01,0.55)
$\beta _1$ (CKD, 28)	-0.25(-0.39,-0.13)	-0.25(-0.38,-0.13)	-0.25(-0.37,-0.13)
$\beta _2$ (Diabetes, 21)	-0.30(-0.48,-0.13)	-0.30(-0.48,-0.14)	-0.30(-0.48,-0.15)
$\beta _3$ (Glomerular Diseases, 10)	-0.35(-0.66,-0.16)	-0.35(-0.66,-0.17)	-0.35(-0.66,-0.18)
Analysis: Small Intervention Subgroups Left-Out (7 Subgroups in Model Fitting)			
Mean $\beta$	-0.41(-0.75,-0.13)	-0.41(-0.74,-0.13)	-0.4(-0.68,-0.18)
Between-Subgroup SD of $\beta$	0.19(0.02,0.73)	0.18(0.02,0.70)	0.15(0.01,0.47)
$\beta _1$ (Antiplatelets, 3)	-0.39(-1.03,0.34)	-0.39(-1.01,0.33)	-0.39(-0.88,0.14)
$\beta _2$ (DPP-4, 3)	-0.40(-1.04,0.16)	-0.40(-1.01,0.14)	-0.39(-0.89,0.04)
$\beta _3$ (Immunosuppressants, 9)	-0.47(-0.92,-0.23)	-0.47(-0.94,-0.24)	-0.46(-0.87,-0.24)
$\beta _4$ (Modify Blood Pressure, 7)	-0.45(-0.84,-0.18)	-0.44(-0.83,-0.17)	-0.43(-0.78,-0.18)
$\beta _5$ (RASB vs CCB, 4)	-0.41(-0.81,-0.06)	-0.41(-0.79,-0.06)	-0.41(-0.75,-0.14)
$\beta _6$ (RASB vs Control, 21)	-0.50(-0.82,-0.25)	-0.49(-0.82,-0.24)	-0.47(-0.75,-0.23)
$\beta _7$ (SGLT-2, 4)	-0.25(-0.49,0.01)	-0.25(-0.49,0.00)	-0.27(-0.48,-0.07)

Intervention subgroup names: *DPP-4 *Dipeptidyl peptidase 4 inhibitor, *RASB *Renin-angiotensin system blockers, *CCB *Calcium channel blockers, *SGLT-2 *Sodium-glucose Cotransporter-2 inhibitors, *SD *Standard deviation

**Fig. 1:**
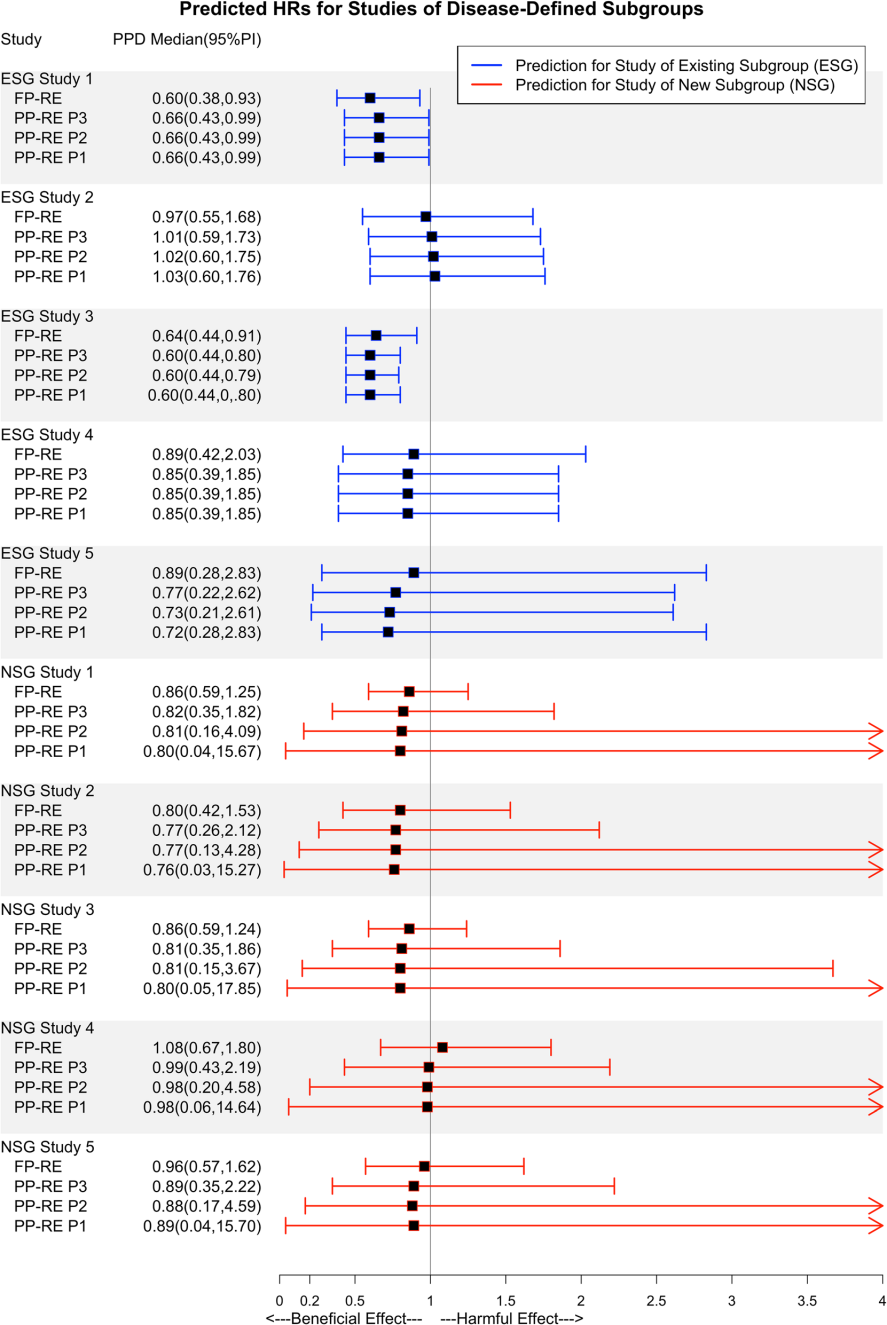
Posterior predictive median and 95% interval are summarized. FP-RE: Full-pooling random effects. PP-RE: Partial-pooling random effects. P1: Diffuse priors used in fitting the PP-RE model. P2: Constrained priors set 1 in fitting the PP-RE model. P3: Constrained priors set 2 (narrowest) in fitting the PP-RE model. Studies listed are described further in Additional file 1. The “ESG” (existing subgroup) studies were used for model fitting. The “NSG” (new subgroup) studies were left-out of model fitting

**Fig. 2:**
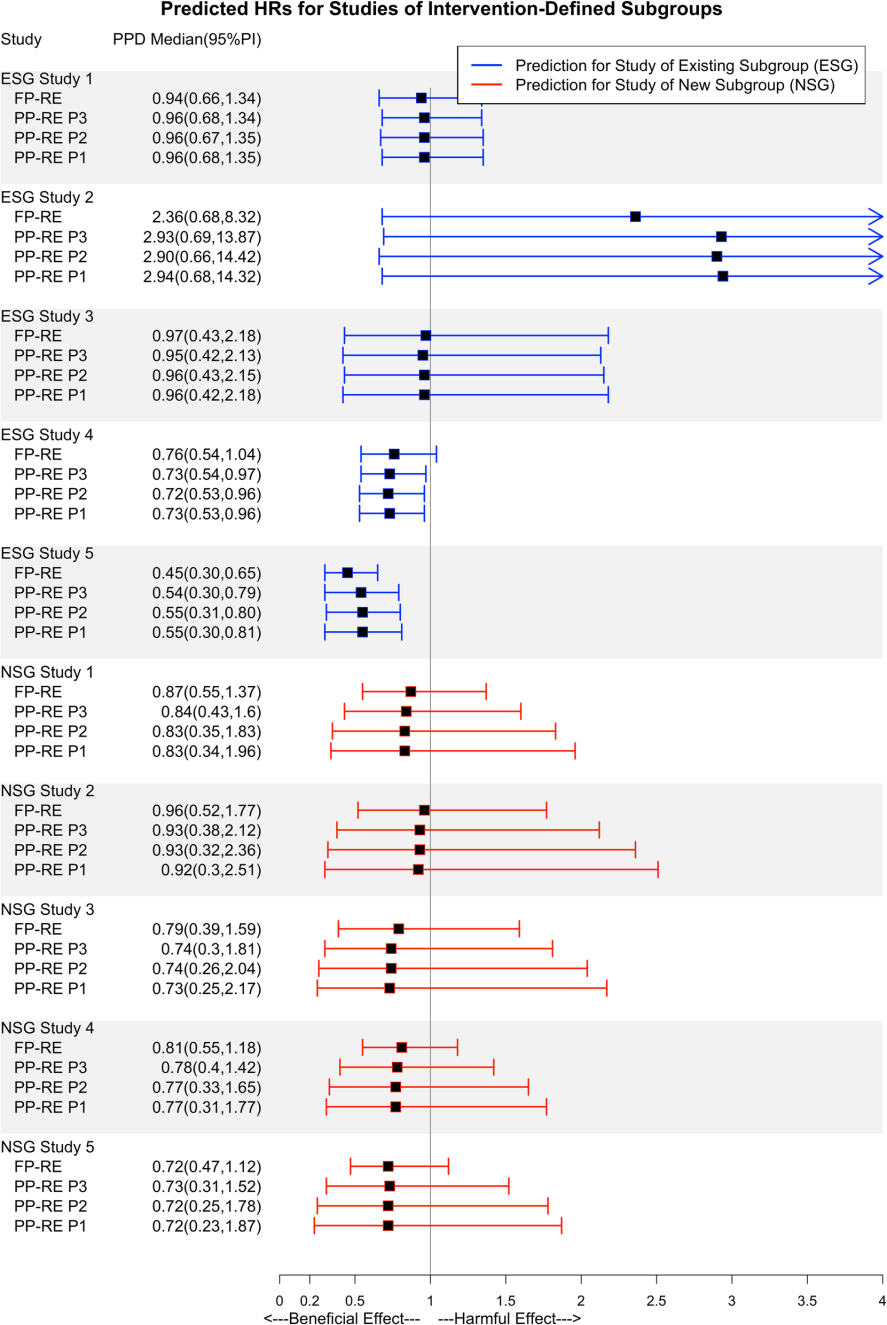
Posterior predictive median and 95% interval are summarized. FP-RE: Full-pooling random effects. PP-RE: Partial-pooling random effects. P1: Diffuse priors used in fitting the PP-RE model. P2: Constrained priors set 1 in fitting the PP-RE model. P3: Constrained priors set 2 (narrowest) in fitting the PP-RE model. Studies listed are described further in Additional file 1. The “ESG” (existing subgroup) studies were used for model fitting. The “NSG” (new subgroup) studies were left-out of model fitting

Figures 1 and 2 display and illustrate the implications of the choice of priors on prediction for trials of a new subgroup or an existing subgroup. A subset of trials is displayed in the figures to be concise, and the remaining results are displayed in Additional file 1: Tables 7-12. Firstly, consider the trials of novel subgroups. For every study, the PP-RE model resulted in wider PPDs than the FP-RE model. When there were fewer subgroups, predictive distributions for left-out studies were excessively and unrealistically wide when using completely diffuse priors under the PP-RE model. The use of constrained priors, especially those motivated by domain-specific reasoning (P3), resulted in PPDs which were narrowest among those obtained, but still wider than those under the FP-RE model with diffuse priors. Increasingly constrained priors resulted in more realistic uncertainty in HRs relative to the use of diffuse priors. When predicting for a trial of a novel intervention class (Fig. 2), where more subgroups were available for model-fitting, PPDs were narrower under the PP-RE approach (contrast PPDs in Fig. 1 relative to Fig. 2). This could be because of improved inferential precision for parameters associated with between-subgroup variability when more subgroups are present. These results indicate the PP-RE model may be more suitable for prediction to induce an appropriate degree of added uncertainty in predicting a clinical effect in a trial meaningfully different than those used to evaluate the surrogate. However, these results also suggest that PPDs can be excessively wide due to overly diffuse and unrealistic priors and not due to the true quality of the surrogate or its applicability to a new setting. Next, when trials were of a subgroup available for model fitting, the summaries of PPDs under the PP-RE model were more robust to the choice of priors relative to prediction for studies of a new subgroup (even for subgroups with few trials). In our setting, predictive distributions were also similar in width under the PP-RE relative to FP-RE model (evidenced by the 2.5^th^ and 97.5^th^ percentiles). The PP-RE model may thus increase accuracy and precision in prediction of clinical effects for future trials of existing subgroups over use of the FP-RE model by allowing subgroup-specific meta-regression parameters.

## Discussion

Trial-level surrogate endpoint evaluations are often performed on collections of heterogeneous clinical trials. Standard methodology that yields estimates of a single set of meta-regression parameters may not be appropriate when trials meaningfully differ across pre-specified subgroups, and may also provide unrealistic precision in prediction of clinical effects in new studies that differ from those used to evaluate the surrogate. In this paper, we explored a class of models we refer to as “partial-pooling” models, where subgroup-specific meta-regressions are assumed, and yet between-subgroup distributions facilitate data adaptive information sharing across subgroups. Partial-pooling models provide a framework both for prediction of treatment effects on the clinical endpoint for a trial that meaningfully differs (is of a new subgroup) from those used for the surrogate evaluation itself and for prediction of future studies of an existing subgroup. There are various challenges in the implementation of a partial-pooling approach, such as the choice of priors and distribution for the true treatment effects on the surrogate. We conducted analyses to help guide such decision making.

Under the scenarios considered (e.g., unless there are a large number, exceeding at least 30, of large trials within a given subgroup), our analyses indicated that fitting separate models for surrogate endpoint evaluation within subgroups (no-pooling) can result in excessive uncertainty in posteriors. We found that partial-pooling methods can be a practical solution with noteworthy benefits (we saw improved precision in posteriors with limited bias due to information sharing in our analyses). If interest is in inference for subgroup-specific meta-regression posteriors, our results showed key differences in interpretations when using fixed versus random effects under the partial-pooling approach. In our analyses, the partial-pooling fixed effect variant produced downward bias in the meta-regression slope in subgroups of trials where the surrogate was strong, which translated to more biased prediction. The partial-pooling random effects approach did not produce such biases in subgroups where the surrogate was strong. We also did not see noteworthy biases under the partial-pooling random effects approach when the Gaussian distributional assumption of the true treatment effects on the surrogate was definitively violated.

A key theme of our results is that posterior distributions of the meta-regression parameters within each subgroup under the partial-pooling random effects model were robust to a degree of narrowing of priors on between-subgroup parameters. Similarly, inferences which apply the meta-regressions fit under the partial pooling model to estimate the posterior predictive distribution for the treatment effect on the clinical endpoint in a new trial were robust to the prior distributions when the new trial belonged to one of the same subgroups included when fitting the meta-regression. Conversely, however, inferences to a new trial which did not belong to one of the subgroups of the prior trials could be highly dependent on the prior distributions, especially for priors on the between subgroup standard deviations of the meta-regression parameters. Notably, when highly diffuse priors were used, the posterior predictive distributions for the new trial exhibited very high dispersion, indicating poor ability to extend the relationship between the treatment effects on the surrogate and clinical endpoints from the previous trials to the new trial. The extent to which the choice of priors influenced dispersion of posterior predictive distributions for a trial of a new subgroup was greater when there were fewer subgroups used in model fitting (e.g., if there were 3 as opposed to 7 subgroups, as in our analyses). This suggests that when fitting partial-pooling models, not only the use of overly constrained, but also the use of overly diffuse priors can unduly influence certain predictive analyses, and it is thus important to consider a strategy to identify more practical priors.

These quantitative findings are consistent with the general concept that the relationship between treatment effects on the surrogate and clinical endpoints observed in previously conducted trials can be reasonably applied to a new trial if at least one of the following three conditions hold: 1) there is strong evidence for a high-quality surrogate with a lack of heterogeneity in performance across a large number of subgroups representing an exhaustive array of intervention types and disease sub-classifications; 2) the new trial can be viewed as a member of the same subgroups used to evaluate the surrogate; 3) subject matter knowledge is sufficiently strong to support informative prior distributions, which mitigate heterogeneity in the meta-regression parameters between subgroups. This third condition appears related to the stress regulatory agencies place on the strength of evidence for a strong biological relationship between the surrogate and clinical endpoints. If the new trial is evaluating a novel treatment or disease subtype which is fundamentally distinct from any of the previous subgroups of trials, and subject matter knowledge cannot rule out heterogeneity in the meta-regression parameters between subgroups, application of the relationship between the surrogate and clinical endpoints observed in the prior trials to the new trial is tenuous. Of course, priors which drive the applicability of the meta-regression for prediction to a trial of a new subgroup can be tuned with multiple considerations in mind. In one regard, even without strong subject matter knowledge, basic logic can be used to narrow priors to some degree (such as for the meta-regression intercept, a log hazard ratio in our case, which is a commonly used metric and need not be expected to vary excessively). On the other hand, priors could be further constrained if there is strong subject matter knowledge indicating to do so, ideally from multiple stakeholders. Key is that the use of completely diffuse priors is likely to be highly impractical when employing partial-pooling models for surrogate evaluation, and the applicability of the surrogate should not depend on the excessive uncertainty imposed by the use of such priors as opposed to those that are realistic according to sound subject matter reasoning.

A noteworthy implication of our findings is that use of a partial-pooling model on a diverse collection of studies may be more useful than highly targeted surrogate evaluations on small subsets of studies. For example, there have been many evaluations of surrogates such as tumor response or progression free survival for highly specific tumor types in cancer [19–22]. However, there may be insufficient data in such settings to truly infer the quality of the surrogate. Partial-pooling models (with appropriately defined priors) fit to data sets with more tumor types, for example, may yield more useful information than fitting separate models within the small subgroups.

There are potential limitations to our analyses and findings. The use of Bayesian methods for surrogate evaluation is computationally demanding and we thus considered a limited number of scenarios in our application and simulation analyses. There may also be many additional distributions that could provide further benefit over the Gaussian or fixed-effects approaches we considered. For example, Bujkiewicz et al. showed potential benefits of using a t-distribution for certain terms [8]. Other strategies to refine priors may also be appropriate in other disease settings. Our analyses and discussion are embedded within the context where we initiate the analysis by assuming (through our priors) there may be some heterogeneity in the meta-regression across subgroups, but that priors on terms related to between-subgroup heterogeneity can be narrowed to some degree to ensure the inference is not unduly influenced by unrealistically wide priors. An alternative approach may be to use priors which, to some degree, induce the assumption that there is no between-subgroup heterogeneity in the quality of the surrogate to start the analysis, forcing the data to provide strong evidence for heterogeneity for the meta-regression posteriors to differ at all across subgroups. For example, spike and slab priors could be considered in future work, if the use of such priors aligns with the analytical goals in a given surrogate evaluation.

It is also important to note that there are many approaches to trial-level surrogate endpoint evaluation. For example, Buyse et al. have proposed joint models that can be fit in a single-stage analysis to simultaneously estimate within and between-study surrogacy metrics [23]. While joint modeling strategies have a number of advantages, their uptake appears less common than two-stage approaches in practice [9]. Other authors have also used network meta-regression strategies for surrogate endpoint evaluations on collections of heterogeneous studies [24]. Finally, within the context of evaluating whether there is heterogeneity in trial-level associations, alternative model structures may be useful depending on the ultimate scientific question. For example, one might consider a single linear regression with interaction terms. One potential drawback to such an approach is that with increasing trial-level factors (e.g., subgroups), such models become increasingly complex, potentially over-parameterized, and may pose challenges for non-statisticians to interpret. On the other hand, an advantage of the partial-pooling approaches discussed is that these maintain the linear regression structure within subgroups, which is again an approach that is already familiar to many investigators.

## Conclusions

The methods discussed in this paper are applicable to the two-stage approach often used to establish the trial-level validity of a surrogate endpoint. Because establishing trial-level surrogacy requires a collection of clinical trials, analysts are often confronted with limited data. A strategy to overcome such data limitations is to incorporate a broad collection of studies with various disease and therapy sub-categories. However, analyses on such data in, for example, chronic kidney disease has encouraged regulatory agencies to question whether surrogate performance varies across pre-specified and clinically motivated subgroups of trials defined by disease or intervention classes. Analyses requiring sub-dividing available trials into subgroups will only exacerbate issues associated with model fitting on small amounts of data. We performed analyses that showed that partial-pooling modeling approaches may improve the potential to infer the quality of the surrogate within subgroups of trials even on limited datasets. However, our analyses also showed that even diffuse priors used for partial-pooling analyses can strongly influence the perceived quality of the surrogate as well as the ability to predict the treatment effect on the clinical endpoint. We discussed strategies that can be used to constrain priors used for the analysis to obtain more realistic estimates of key parameters for surrogate endpoint evaluation. Ultimately, analyses of a surrogate endpoint could result in appropriately expanding the feasibility of trials in an entire disease area, or could lead to the use of an endpoint that is not ultimately useful for patients. Partial-pooling models should be considered for surrogate endpoint evaluation on heterogeneous collections of trials, but the choice of a given model and priors to implement the model should be handled rigorously.

### Supplementary Information

**Additional file 1.**

## Data Availability

Data restrictions apply to the data used for the application analyses presented, for which we were given access under license for this manuscript. These data are not publicly available due to privacy or ethical restrictions. The programs used to generate data used for the purposes of the simulation study is provided in the supplemental materials.

## Abbreviations

CKD: Chronic kidney disease
GFR: Glomerular filtration rate
RE: Random effects
FP: Fixed-effects
FP: Full-pooling
NP: No-pooling
PP: Partial-pooling
PPD: Posterior predictive distribution
DM: Diabetes mellitus
GN: Glomerular disease
CVD: Cardiovascular disease
IG: Inverse-gamma
