# Supplementary material for: Comparing Bayesian hierarchical meta-regression methods and evaluating the influence of priors for evaluations of surrogate endpoints on heterogeneous collections of clinical trials
Source: BMC Med Res Methodol. 2024 Feb 16;24:39. doi: 10.1186/s12874-024-02170-0 (PMC10870489; doi:10.1186/s12874-024-02170-0)
Supplement: Supplementary file 1 — Additional file 1. [file 12874_2024_2170_MOESM1_ESM.pdf]

1 Intervention-Defined Subgroups in the CKD-EPI Data

Table 1: Summarizing Intervention-Defined Subgroups for Application Analyses

Intervention Subgroup	Number of Studies	Used For
Antiplatelet	3	Model fitting/Prediction
Dipeptidyl peptidase 4 (DPP-4) Inhibitors	3	Model fitting/Prediction
Immunosuppression	9	Model fitting/Prediction
Blood pressure lowering agent	7	Model fitting/Prediction
RASB vs CCB	4	Model fitting/Prediction
RASB vs Control	21	Model fitting/Prediction
Sodium-glucose Cotransporter-2 (SGLT2) Inhibitors	4	Model fitting/Prediction
Albuminuria targeted therapy	1	Prediction Only
Allopurinol	2	Prediction Only
Endothelin receptor Antagonist	1	Prediction Only
Glucagon-like peptide-1 receptor Agonist	2	Prediction Only
Intensive Glucose Intervention	1	Prediction Only
Low vs usual diet	2	Prediction Only
Mineralocorticoid receptor Antagonist	2	Prediction Only
Nurse-coordinated care	2	Prediction Only
RASB+CCB vs Control	1	Prediction Only
Statins and Ezetimibe	1	Prediction Only

RASB: Renin angiotensin system blockers; CCB: Calcium chanel blockers.

2 Constrained Priors for PP-RE Model Fitting

As discussed in the main manuscript, we considered two sets of constrained priors applied when fitting the PP-RE model in our application analyses. For Set 1, we used an iterative procedure to find the narrowest priors for $\sigma_\alpha, \sigma_\beta, \gamma_e, \gamma_s, \sigma_s$, after which further narrowing resulted in altered subgroup-specific meta-regression posteriors for the meta-regression intercept, slope, or residual SD, relative to those obtained from fully diffuse priors. "Altered" was defined as when either the median, 2.5^{th}, or 97.5^{th} posterior percentiles differed by 0.05 or more between the narrower or diffuse priors. The priors obtained for each of these terms after utilizing this procedure were as follows. We note again that narrowing of priors related to the distribution of true treatment effects on the surrogate is of less importance to those

related to the meta-regression.

$$\gamma_s \sim \text{half} - \text{normal}(0, 2^2) \tag{1}$$

$$\gamma_e \sim \text{half} - \text{normal}(0, 2.25^2) \tag{2}$$

$$\sigma_\alpha \sim \text{half} - \text{normal}(0, 0.7^2) \tag{3}$$

$$\sigma_\beta \sim \text{half} - \text{normal}(0, 0.7^2) \tag{4}$$

$$\sigma_s \sim \text{half} - \text{normal}(0, 0.7^2) \tag{5}$$

For our "Set 2" of constrained priors, we further constrained the priors displayed above based on subject-specific reasoning. We note that these priors may have been further constrained and could still have allowed more between-subgroup variation in any given subgroup-specific parameter than we may expect in practice. Nonetheless, these priors provided important insight into the influence of choices of priors on results of subgroup-specific surrogate endpoint evaluation. The specific priors chosen were as follows. We later provide histograms to visually contrast these priors for the meta-regression slope with the corresponding diffuse priors.

$$\gamma_s \sim \text{half} - \text{normal}(0, 1.75^2) \tag{6}$$

$$\gamma_e \sim \text{half} - \text{normal}(0, 1.45^2) \tag{7}$$

$$\sigma_\alpha \sim \text{half} - \text{normal}(0, 0.2^2) \tag{8}$$

$$\sigma_\beta \sim \text{half} - \text{normal}(0, 0.4^2) \tag{9}$$

$$\sigma_s \sim \text{half} - \text{normal}(0, 0.8^2) \tag{10}$$

In terms of rationale for choosing priors, first consider σ_α. Imagine the true between-subgroup mean intercept is 0 for illustrative purposes. If we assume σ_α is drawn from a half-normal(0,0.2), 75% of the distribution is less than approximately 0.23. A normal distri-

bution (for the within-subgroup intercepts) centered at zero with SD 0.23 will have 50% of the data between -0.15 and 0.15, a range (for a log-hazard ratio) that already likely exceeds that which we would expect for subgroup specific mean intercepts. Yet, this distribution still allows 50% of the data to be beyond those bounds. Furthermore, by drawing from half-normal(0,0.2^2) for σ_α and $N(0, 3^2)$ for μ_α, this implies the subgroup-specific priors for the meta-regression intercept have 50% of the data between -2 and 2, again a range that exceeds expectation when the true treatment effect on the surrogate is the null effect (as a reminder, in the CKD context considered, the intercept represents a log-hazard ratio). Thus, these priors are still quite diffuse, but are considerably more constrained than diffuse priors.

For σ_β, first imagine the true between-subgroup mean slope is -0.3 (close to the posterior median of the between-subgroup mean in the application analysis when disease-defined subgroups were used, as an example). If we assume σ_β is drawn from a half-normal(0,0.4^2), 75% of SDs will be less than about 0.46. A normal distribution centered at -0.3 with SD 0.46 will have 50% of the data between about 0 and -0.6, representing a wide range of meta-regression slopes varying from twice as strong as what we might expect (-0.6) to the null slope (0). Even still, the prior on σ_β allows for even larger SDs. This thus allows more variation in subgroup specific mean slopes than we would likely expect in reality. Furthermore, a prior on μ_β of $N(0, 3^2)$ implies the subgroup specific slopes have a prior with 50% of the data between -2 and 2, again much wider than we would expect in reality yet providing more constraint over completely diffuse priors.

The prior on σ_s was half-normal(0,0.8^2). This prior is more diffuse than those listed above so as not to constrain how much variation there is in mean true effects on the surrogate across subgroups. We wanted to allow some additional heterogeneity in the mean true treatment effect on the surrogate across subgroups to allow the effect sizes (x-axis variable) to be determined by the location of the center observed in past trials. However, with the prior

placed on μ_s, this combination of priors implies at least 50% of the subgroup-specific mean true effects on the surrogate will be between -2 and 2, a much wider range than we would ever expect in reality given that among the majority of studies, the estimated effect on chronic slope is less than 2 (in mL/min/1.73m^2 per year) (indicated treatment benefit) and at minimum an effect on the surrogate of -1 (indicating treatment harm).

Imagine the between-subgroup mean meta-regression residual SD is 0.1, then letting γ_e have a half-normal prior with SD 1.45 implies 75% of SDs are below 1.65. An SD of 1.65 implies 50% of subgroup specific meta-regression residual-SDs are between 0.03 and 0.30, a range that currently exceeds expectation and allows for a range of both highly accurate prediction within a subgroup (0.03) to modestly inaccurate prediction within a subgroup (0.30). Of course, because the prior still allows subgroup-specific residual SDs above 0.30 with a reasonably high probability, this arrangement still facilitates subgroups where the surrogate is yet even weaker than expected. Note also that the prior for γ_s in Set 2 was only modestly constrained over Set 1.

The discussion above illustrates the rationale used to choose priors for our "Constrained Set 2." Of course, there are many strategies that could be used to identify priors which provide reasonable constraint to the inference based on domain-specific reasoning. It may also be helpful to employ a prior elicitation process, possibly with the help of clinicians.

In terms of the meta-regression slope, in particular, consider the figures below for further intuition. We display histograms for the priors for the between-subgroup SD for the subgroup-specific meta-regression slopes (σ_β) and the between-subgroup mean slopes (μ_β), and then display figures that represent the implied priors, based on these first two terms, for the within-subgroup meta-regression slopes (β_j for $j = 1, \ldots, C$ subgroups). Figures 1 and 2 are for the between-subgroup SDs.

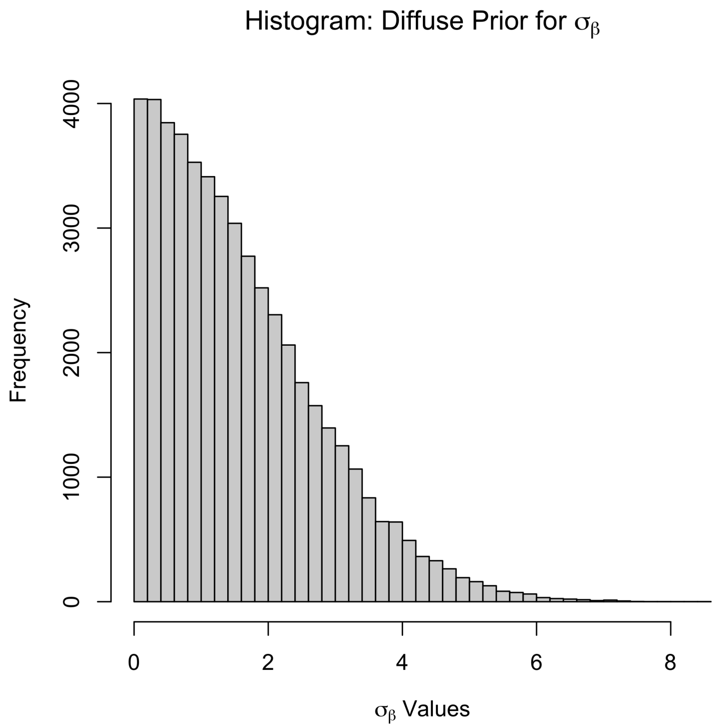

Figure 1: Fully diffuse prior for the between-subgroup SD of the subgroup-specific meta-regression slopes.

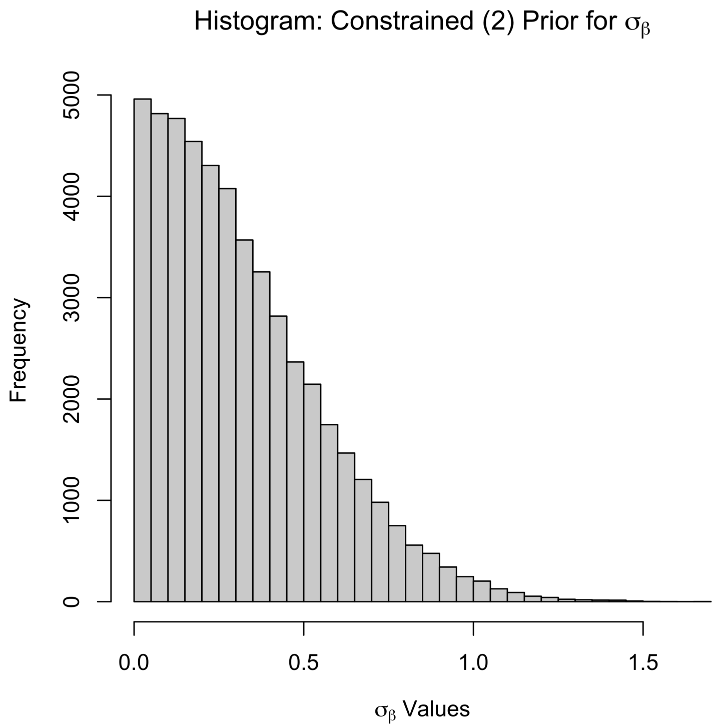

Figure 2: Constrained prior from "Set 2" for the between-subgroup SD of the subgroup-specific meta-regression slopes.

Note that the fully diffuse prior for σ_β is considerably wider than that of the constrained prior. However, values in excess of 1, say, for the SD σ_β are highly unrealistic in our application analyses considering that the subgroup-specific meta-regression slopes in our application seem to be centered at around -0.3 to -0.5 and are very unlikely to exceed 0 or be below -1 for any subgroup. The diffuse prior would still facilitate a posterior for this term with probability mass above 1 if the data strongly suggested such high variation in the meta-regression slope across subgroups.

Meanwhile, we only slightly constrained the between-subgroup mean meta-regression slope prior (μ_β), and the posteriors for this term are displayed in Figures 3 and 4. Clearly, considering we expect the subgroup specific meta-regression slopes to be between about 0 and -1, both priors could be considered diffuse.

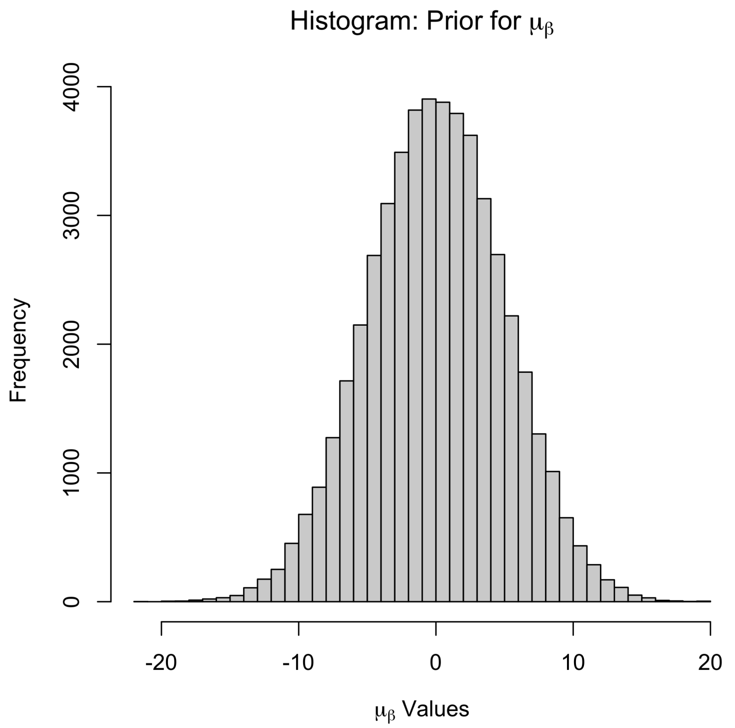

Figure 3: Fully diffuse prior for the between-subgroup mean of the subgroup-specific meta-regression slopes.

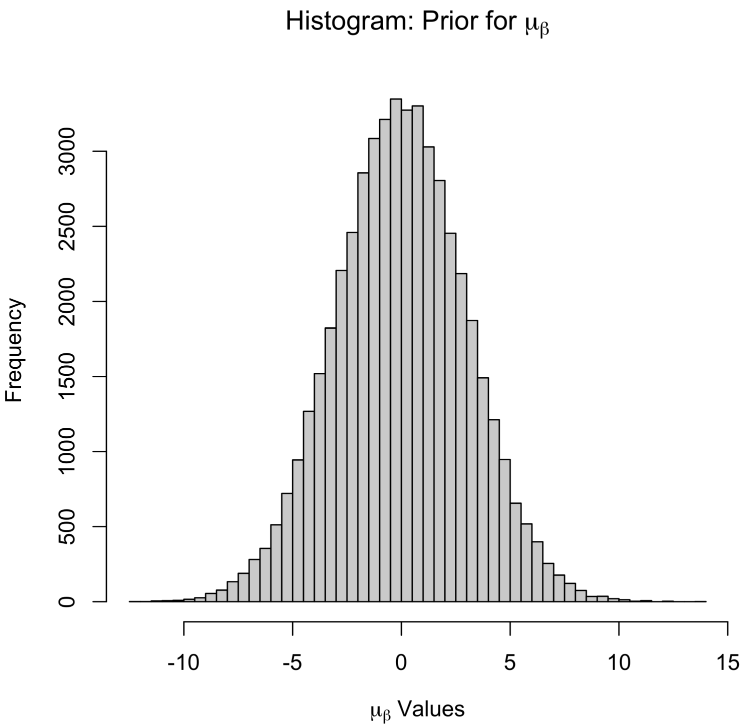

Figure 4: Constrained prior from "Set 2" for the between-subgroup mean of the subgroup-specific meta-regression slopes.

Finally, recall that for each subgroup indexed by j, the subgroup-specific meta-regression slopes are assumed to follow the normal distribution $\beta_j \sim N(\mu_\beta, \sigma_\beta^2)$ under the partial-pooling models. The above set of diffuse and constrained priors for μ_β and σ_β displayed above translate to the implied priors for β_j displayed in Figures 5 and 6, respectively.

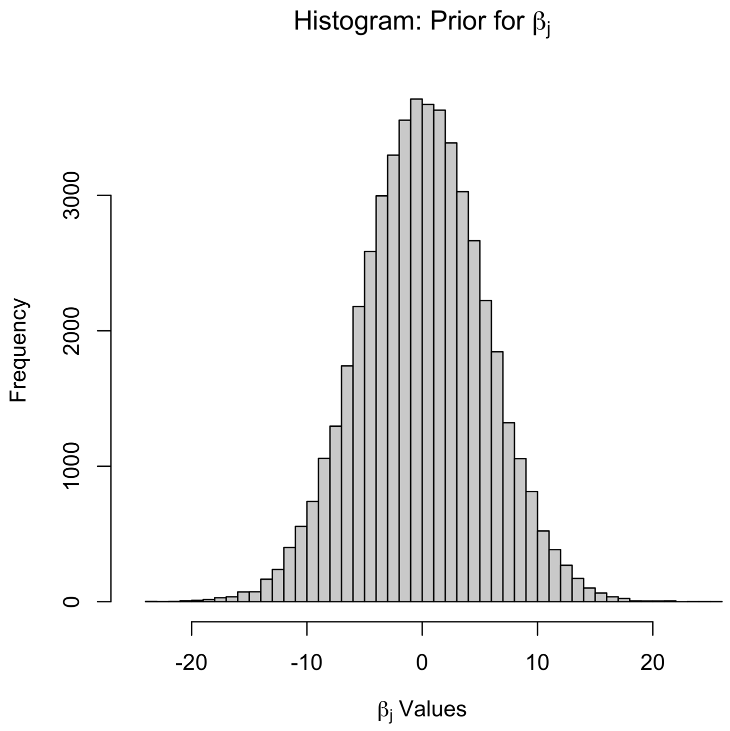

Figure 5: Fully diffuse prior for each subgroup-specific meta-regression slope.

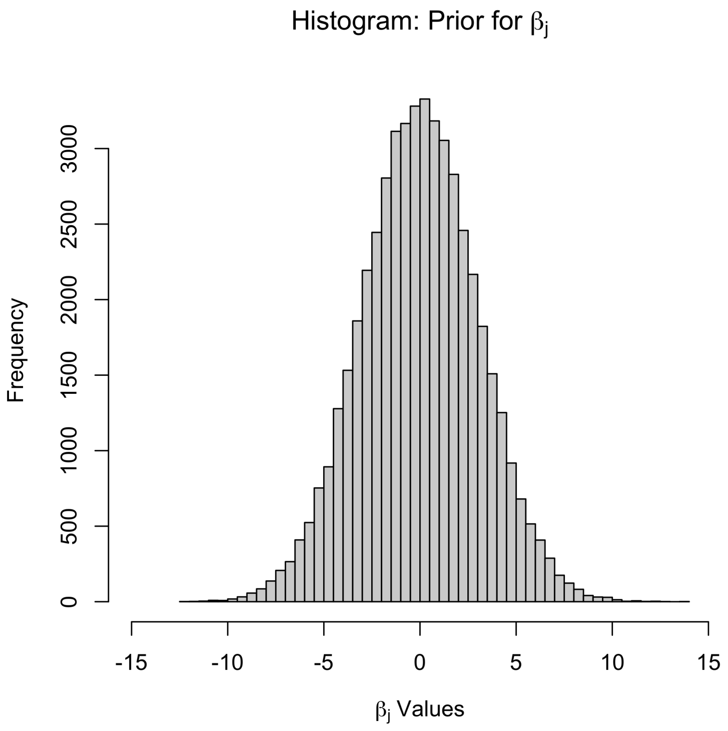

Figure 6: Constrained prior from "Set 2" for each subgroup-specific meta-regression slope.

One can see that the priors implied for subgroup-specific slopes are indeed narrower when using the constrained rather than diffuse priors for μ_β and σ_β. However, the prior for β_j under the constrained priors is still substantially wider than we would expect the posterior to be. As such, while the prior for σ_β can be constrained considerably to be more realistic based on subject-matter reasoning, doing so still facilitates wide priors for the subgroup-specific terms of primary interest.

3 Generating Posterior Predictive Distributions

In the main manuscript, we described the process for generating posterior predictive distributions (PPDs) for the true treatment effects on the clinical endpoint in our simulation study. Here, we describe the procedures used to generate PPDs for estimated effects on the

clinical endpoint, taking into account the estimation error for the treatment effect estimates within a study as well as uncertainty in the meta-regression parameters used to map the estimated effect on the surrogate to that of the clinical endpoint. First, consider prediction under the full-pooling random-effects model (FP-RE). Note that the following procedure would be used under the assumption that treatment effect on the clinical endpoint is being predicted for a trial that is fully-exchangeable with the studies used for model fitting. Recall the model notation used in the main manuscript, which we display again here:

$$(\widehat{\theta}_{1j}, \widehat{\theta}_{2j})' \sim N((\theta_{1j}, \theta_{2j})', \Sigma_j),$$

$$\theta_{2j} \sim N(\mu_s, \sigma_s^2), \quad \text{and} \quad \theta_{1j}|\theta_{2j} \sim N(\alpha + \beta\theta_{2j}, \sigma_e^2).$$

Under this model, we can write the joint marginal distribution for the pair of the estimated effects on the clinical and surrogate endpoints for trial j as the following, where $\Omega_{1,1} = \sigma_c^2$ is the total between-study variance for the true treatment effect on the clinical endpoint, $\Omega_{2,2} = \sigma_s^2$ is the total between-study variance for the true treatment effects on the surrogate, and $\Omega_{1,2} = \Omega_{2,1} = \rho_b\sigma_c\sigma_s$ where ρ_b is the between-study correlation between the true treatment effects on the two endpoints.

$$(\widehat{\theta}_{1j}, \widehat{\theta}_{2j})' \sim N((\alpha + \beta\mu_s, \mu_s)', \Sigma_j + \Omega).$$

The joint distribution above implies that we can write the conditional distribution of the estimated effect on the clinical endpoint on the estimated effect on the surrogate endpoint $(\widehat{\theta}_{1j}|\widehat{\theta}_{2j} = a)$ as a normal distribution with mean $E(\widehat{\theta}_{1j}|\widehat{\theta}_{2j} = a) = \alpha + \beta\mu_s + \frac{(\widehat{r}_j SE(\widehat{\theta}_{1j})SE(\widehat{\theta}_{2j}) + \rho_b\sigma_c\sigma_s)}{\sqrt{(SE(\widehat{\theta}_{1j})^2 + \sigma_c^2)(SE(\widehat{\theta}_{2j})^2 + \sigma_s^2)}}(\frac{\sqrt{SE(\widehat{\theta}_{1j})^2 + \sigma_c^2}}{\sqrt{SE(\widehat{\theta}_{2j})^2 + \sigma_s^2}})(a - \mu_s)$ and with variance $var(\widehat{\theta}_{1j}|\widehat{\theta}_{2j} =$

$a) = (1 - [\frac{(\widehat{r}_j SE(\widehat{\theta}_{1j})SE(\widehat{\theta}_{2j}) + \rho_b \sigma_c \sigma_s)}{\sqrt{(SE(\widehat{\theta}_{1j})^2 + \sigma_c^2)(SE(\widehat{\theta}_{2j})^2 + \sigma_s^2)}}]^2)(SE(\widehat{\theta}_{1j})^2 + \sigma_c^2)$. For each study, there would be a single standard error for the estimated effect on the clinical endpoint, a single standard error for the estimated effect on the surrogate endpoint, a single estimated within-study correlation, and a single estimated treatment effect on the surrogate endpoint. We would thus obtain a posterior predictive distribution by taking m draws from the normal distribution displayed above, one for each mcmc draw obtained in model fitting. For each separate draw, we would instead use $\alpha^m, \beta^m, \mu_s^m, \rho_b^m, \sigma_c^m, \sigma_s^m$, denoting separate draws taken from the posterior for each respective parameter obtained through model fitting. If using the partial-pooling random effects model for prediction of a clinical endpoint in a new trial, but of an existing subgroup used for model fitting, we would use the same distribution displayed above, but we would use the m mcmc draws from subgroup-specific meta-regression posteriors estimated. For example, if predicting a clinical effect for a trial of subgroup l, we would instead use $\alpha_l^m, \beta_l^m, \mu_{sl}^m, \rho_{bl}^m, \sigma_{cl}^m, \sigma_{sl}^m$ in the normal distribution displayed above.

The process for generating PPDs for a new study also of a new subgroup (not a subgroup used for model fitting) under the partial-pooling random effects model differs in a key way. For example, this procedure would be applied when generating a PPD for a trial in an intervention class not present in the studies used to evaluate the surrogate. In the CKD setting, as an example, this could have been for prediction in a trial evaluating an SGLT-2 inhibitor if no such trial evaluating this therapy was available for the meta-analysis of the surrogate. In this case, note that within a given subgroup indexed by i, we can write the conditional distribution for the estimated effect on the clinical endpoint within that subgroup as normal distribution with mean $E(\widehat{\theta}_{1ij}|\widehat{\theta}_{2ij} = a) = \alpha_i + \beta_i \mu_{si} + \frac{(\widehat{r}_{ij} SE(\widehat{\theta}_{1ij})SE(\widehat{\theta}_{2ij}) + \rho_{bi} \sigma_{ci} \sigma_{si})}{\sqrt{(SE(\widehat{\theta}_{1ij})^2 + \sigma_{ci}^2)(SE(\widehat{\theta}_{2ij})^2 + \sigma_{si}^2)}}(\frac{\sqrt{SE(\widehat{\theta}_{1ij})^2 + \sigma_{ci}^2}}{\sqrt{SE(\widehat{\theta}_{2ij})^2 + \sigma_{si}^2}})(a - \mu_{si})$ and with variance

$var(\widehat{\theta}_{1ij}|\widehat{\theta}_{2ij} = a) = (1 - [\frac{(\widehat{r}_{ij}SE(\widehat{\theta}_{1ij})SE(\widehat{\theta}_{2ij}) + \rho_{bi}\sigma_{ci}\sigma_{si})}{\sqrt{(SE(\widehat{\theta}_{1ij})^2 + \sigma_{ci}^2)(SE(\widehat{\theta}_{2ij})^2 + \sigma_{si}^2)}}]^2)(SE(\widehat{\theta}_{1ij})^2 + \sigma_{ci}^2)$. Again, many of the input (standard errors, within-study correlation, and the effect estimate on the surrogate) necessary to take draws from this normal distribution are estimated or approximated within the trial where prediction is being applied. If the lth subgroup was not available for model fitting, however, than estimates of $\alpha_l, \beta_l, \mu_{sl}, \rho_{bl}, \sigma_{cl}, \sigma_{sl}$ will not be available directly through the posteriors obtained in fitting the PP-RE model. Under the PP-RE model, however, we will have obtained draws from posterior distributions for between-subgroup parameters from which $\rho_{bl}, \sigma_{cl}, \sigma_{sl}, \alpha_l, \beta_l, \mu_{sl}$ can be drawn (e.g., we can take draws from $N(\beta, \sigma_\beta^2)$ using the posteriors obtained for the population (between-subgroup) parameters β, σ_β to obtain a posterior distribution for β_l of a new subgroup). This then defines how we obtain PPDs for trials of a new subgroup. For each mcmc draw obtained in model fitting, we obtain m draws for each of $\alpha_l^m, \beta_l^m, \mu_{sl}^m, \rho_{bl}^m, \sigma_{cl}^m, \sigma_{sl}^m$ by drawing from the relevant between-subgroup distributions for which we have posteriors obtained by fitting the PP-RE model. Each of these draws are then used to take a corresponding draw from the conditional normal distribution displayed above for the PPD. Because the process begins by drawing subgroup-specific parameters from population-subgroup distributions, there is an added degree of uncertainty in this prediction as opposed to the process used to generate a PPD under the FP-RE model. Again, this is intended to reflect the additional uncertainty inherent to generating model-based prediction based on a surrogate that was not vetted for the subgroup represented by the new trial.

4 Supplemental R and RStan Code

4.1 Rstan Code for the PP-RE Model

The following code can be used to implement the partial-pooling random-effects model with diffuse priors. We make note of the locations, in code, where specific priors can be altered to reproduce results under use of the constrained priors Set 1 and Set 2 discussed for our analyses.

```
data{
int nStudies; \\Total number of studies used for the analysis.
vector[nStudies] Sur1Est; \\Vector of estimated treatment effects on
the surrogate.
vector[nStudies] Sur1SE; \\Vector of standard errors of estimated
treatment effects on the surrogate.
vector[nStudies] ClnEst;\\Vector of estimated treatment effects
on the clinical endpoint.
vector[nStudies] ClnSE; \\Vector of standard errors of estimated
treatment effects on the clinical endpoint.
vector[nStudies] R1Clin; \\Vector of within-study correlation estimates.
int firstIndices[3]; \\A set of indices representing where, in ordered
data, each subgroup of trials begins and ends.
int lastIndices[3];
}


parameters{
matrix[nStudies,2] withinStudyMeans; \\Matrix of paired true treatment
```

```
effects.
real muSur1[3]; \\Vector of subgroup-specific average true surrogate
effects.
real<lower=-10,upper=3> log_sigSur1[3]; \\Vector of log-transformed subgroup
-specific SDs.
real alphaClinonSur1[3]; \\Vector of subgroup-
specific meta-regression intercepts.
real betaClinonSur1[3]; \\ Vector of subgroup-specific meta-regression
slopes.
real<lower=-10,upper=3> log_sigClinonSur1[3]; \\Vector of log-transformed subgroup-
specific error-SDs.
real muOverall; \\Population mean true surrogate effect.
real alphaMean; \\Population mean meta-regression intercept.
real betaMean; \\Population mean meta-regression slope.
real<lower=0.0001,upper=10> sigSqSur1Mean; \\Average of subgroup-specific true
surrogate effect variances.
real<lower=0.0001,upper=10> sigSqClinonSur1Mean; \\Average of subgroup-specific
error-variances.
real<lower=0.0001,upper=10> sigMu; \\Between-subgroup SD of average
true
surrogate effects.
real<lower=0.0001,upper=10> sigAlpha; \\Between-subgroup SD of meta-regression
intercepts.
real<lower=0.0001,upper=10> sigBeta; \\Between-subgroup SD of meta-regression
slopes.
```

```
real<lower=0.0001,upper=10> siglog_sigSur1; \\Between-subgroup SD of log-
transformed true surrogate effect SDs.
real<lower=0.0001,upper=10> siglog_sigClinonSur1; \\Between-subgroup SD of log-
transformed error-SDs.
}

model{
vector[2] myY; \\Iteratively, we define each trial's pair of estimated
treatment effects on both endpoints.
matrix[2,2] myVar; \\Iteratively, we define each trial's within-study
variance-covariance matrix.
for(uu in 1:3){ \\Loop through each subgroup for the subgroup-specific
models.
for(jj in firstIndices[uu]:lastIndices[uu]){ \\Loop through trials within
each subgroup.
  // Set-up within study vector of observed effects.
  myY[1]=ClnEst[jj];
  myY[2]=Sur1Est[jj];
  // Set-up within study covariance matrix.
  myVar[1,1]=ClnSE[jj]^2;
  myVar[1,2]=ClnSE[jj]*Sur1SE[jj]*R1Clin[jj];
  myVar[2,1]=Sur1SE[jj]*ClnSE[jj]*R1Clin[jj];
  myVar[2,2]=Sur1SE[jj]^2;
  //Firstly, define the within-study bivariate Gaussian distribution for
  treatment effect estimates.
```

```
  myY~multi_normal(withinStudyMeans[jj,],myVar);
  //Define the product-normal formulation of the subgroup-specific
  distributions for true treatment effects on the surrogate endpoint
  and for the conditional distribution defining the meta-regression.
  withinStudyMeans[jj,2]~normal(muSur1[uu],exp(log_sigSur1[uu]));
  withinStudyMeans[jj,1]~normal(alphaClinonSur1[uu] +
  betaClinonSur1[uu]*withinStudyMeans[jj,2],exp(log_sigClinonSur1[uu]));
}
\\Specify between-subgroup distributions.
muSur1[uu]~normal(muOverall,sigMu);
alphaClinonSur1[uu]~normal(alphaMean,sigAlpha);
betaClinonSur1[uu]~normal(betaMean,sigBeta);
log_sigSur1[uu]~normal(log(sqrt(sigSqSur1Mean)),siglog_sigSur1);
log_sigClinonSur1[uu]~normal(log(sqrt(sigSqClinonSur1Mean)),
siglog_sigClinonSur1);
}
\\Assign priors for all terms summarized above.
sigSqSur1Mean~inv_gamma(0.0025,0.001);
sigSqClinonSur1Mean~inv_gamma(0.0025,0.001);

muOverall~normal(0,10);
alphaMean~normal(0,5);
betaMean~normal(0,5);

\\ The following five priors were altered to provide the constrained sets 1
```

```
\\ and 2 described in the paper and supplemental materials.
siglog_sigSur1~normal(0,3);
siglog_sigClinonSur1~normal(0,3);
sigAlpha~normal(0,2);
sigBeta~normal(0,2);
sigMu~normal(0,2);
}
```

4.2 R Function for Trial-Level Data Simulation

```
# We used the following function to generate a single data-
# set of trials for each simulation iteration.
# nstudies - The number of studies in an overall collection
# of meta-data used for surrogate evaluation.
# subgroups - The number of subgroups of interest (3 throughout
# our application)
# studperg - The number of studies within each subgroup.
# alpha - A vector of true meta-regression intercepts - one
# specified for each subgroup.
# beta - A vector of true meta-regression slopes - one
# specified for each subgroup.
# musur - A vector of true mean treatment effects on the
# surrogate - one for each subgroup.
# musursd - A vector of true treatment effects on the surrogate SD
```

```
# terms - one for each subgroup.
# errorsd - A vector of meta-regression error-SDs - one
# for each subgroup.

simdat.subgroups2 = function(nstudies, subgroups, studperg, alpha, beta,
musur, musursd, errorsd) {
  # The following three blocks of code (beginning with
  # "loc1", "loc2", "loc3") generate a range of trial-
  # specific standard errors and within-study correlations
  # for trials within each of the three subgroups.
  trial.corrs1 = runif(studperg, min = -0.5,max = -0.2)
  trial.surSE1 = runif(studperg,min=0.1,1)
  trial.CESE1 = runif(studperf,min-0.05,0.25)
  trial.corrs2 = runif(studperg, min = -0.5,max = -0.2)
  trial.surSE2 = runif(studperg,min=0.1,1)
  trial.CESE2 = runif(studperf,min-0.05,0.25)
  trial.corrs3 = runif(studperg, min = -0.5,max = -0.2)
  trial.surSE3 = runif(studperg,min=0.1,1)
  trial.CESE3 = runif(studperf,min-0.05,0.25)
  # The following "between.rep1,2,3" are block-diagonal matrices
  # where each diagonal matrix is a true between-study
  # variance-covariance matrix using the fixed, true
  # simulation parameters. We create one broader block-diagonal
  # matrix for each subgroup.
  between.rep1 = kronecker(diag(studperg),
```

```
matrix(c(errorsd[1]^2 + beta[1]^2*musursd[1]^2, beta[1]*musursd[1]^2,
beta[1]*musursd[1]^2, musursd[1]^2), nrow = 2))
between.rep2 = kronecker(diag(studperg),
matrix(c(errorsd[2]^2 + beta[2]^2*musursd[2]^2, beta[2]*musursd[2]^2,
beta[2]*musursd[2]^2, musursd[2]^2), nrow = 2))
between.rep3 = kronecker(diag(studperg),
matrix(c(errorsd[3]^2 + beta[3]^2*musursd[3]^2, beta[3]*musursd[3]^2,
beta[3]*musursd[3]^2, musursd[3]^2), nrow = 2))
between.rep = as.matrix(bdiag(between.rep1, between.rep2, between.rep3))
# Generate three sets (one per-subgroup) of block diagonal
# matrices for the within-study variance-covariance matrices.
tcovmats1 = as.list(data.frame(t(matrix(c(trial.CESE1^2,
trial.corrs1*trial.surSE1*trial.CESE1, trial.corrs1*trial.surSE1*trial.CESE1,
trial.surSE1^2), nrow = studperg))))
tcovmats2 = as.list(data.frame(t(matrix(c(trial.CESE2^2,
trial.corrs2*trial.surSE2*trial.CESE2,trial.corrs2*trial.surSE2*trial.CESE2,
trial.surSE2^2), nrow = studperg))))
tcovmats3 = as.list(data.frame(t(matrix(c(trial.CESE3^2,
trial.corrs3*trial.surSE3*trial.CESE3,
trial.corrs3*trial.surSE3*trial.CESE3, trial.surSE3^2), nrow = studperg))))
# This loop and the two lines of code directly below creates
# one larger block-diagonal matrix, where the diagonal matrices
# are the within-study variance-covariance matrices across
# trials.
for (t in 1:length(tcovmats1)) {
```

```
  tcovmats1[[t]] = matrix(tcovmats1[[t]], nrow = 2)
  tcovmats2[[t]] = matrix(tcovmats2[[t]], nrow = 2)
  tcovmats3[[t]] = matrix(tcovmats3[[t]], nrow = 2)
}
tcovmats = c(tcovmats1, tcovmats2, tcovmats3)
tcovmats = as.matrix(bdiag(tcovmats))
# Generate nstudies pairs of estimated treatment effects
# on the surrogate and clinical endpoints with one draw
# from a multivariate normal distribution using the
# meta-regression parameters as well as the block-diagonal
# matrices created above.
esteffs = c(rep(c(alpha[1] + beta[1]*musur[1], musur[1]), studperg),
            rep(c(alpha[2] + beta[2]*musur[2], musur[2]), studperg),
            rep(c(alpha[3] + beta[3]*musur[3], musur[3]), studperg)) +
            mvrnorm(1, mu = rep(0, times = 2*nstudies),
            Sigma = (tcovmats + between.rep))
# Separate treatment effects on the clinical and surrogate endpoints.
ClnEst.sim = esteffs[seq(from = 1, to = 2*nstudies - 1, by = 2)]
Sur1Est.sim = esteffs[seq(from = 2, to = 2*nstudies, by = 2)]
# Generate list of data elements for stan.
simdat<<-list(
  nStudies=nstudies,
  Sur1Est=Sur1Est.sim,
  Sur1SE=c(trial.surSE1,trial.surSE2, trial.surSE3),
  ClnEst=ClnEst.sim,
```

```
    ClnSE=c(trial.CESE1,trial.CESE2, trial.CESE3),

    R1Clin=c(trial.corrs1,trial.corrs2, trial.corrs3),

    firstIndices=c(1,studperg+1, 2*studperg + 1),

    lastIndices=c(studperg, 2*studperg, nstudies)

  )

  return(simdat)

}
```

5 Additional Results from Simulation Analyses

Table 2: Exploring Sensitivity of Partial-Pooling Performance Gains

	8 Trials/Subgroup		22 Trials/Subgroup		$\sigma_s^{new} = 0.5 \times \sigma_s^{old}$		$\sigma_s^{new} = 1.5 \times \sigma_s^{old}$		$\sigma_s^{new} = 2 \times \sigma_s^{old}$	
Term	RR_{np}	WR_{np}	RR_{np}	WR_{np}	RR_{np}	WR_{np}	RR_{np}	WR_{np}	RR_{np}	WR_{np}
α_1	1.37	3.51	1.51	1.98	0.93	2.54	1.40	1.93	1.30	1.51
α_2	1.50	4.01	1.67	2.25	1.38	3.21	1.49	1.98	1.42	1.40
α_3	2.12	5.14	1.67	2.00	2.14	3.49	1.46	1.98	1.47	1.60
β_1	1.68	4.55	1.56	2.54	1.12	3.26	1.37	2.47	1.18	1.89
β_2	1.59	4.25	1.49	2.47	1.21	3.16	1.32	2.28	1.43	1.45
β_3	1.79	4.89	1.42	1.98	2.04	3.26	1.46	2.23	1.15	1.46
σ_{e1}	0.83	1.32	1.00	1.14	0.88	1.12	0.95	1.17	0.97	1.19
σ_{e2}	0.98	1.33	1.05	1.09	0.89	1.14	1.02	1.15	1.00	1.15
σ_{e3}	1.31	1.36	0.74	1.03	1.13	1.17	0.88	1.07	0.93	1.04
R^2 1	0.75	1.02	0.84	1.05	0.72	1.00	0.81	1.03	0.81	1.00
R^2 2	0.84	1.04	1.20	1.08	0.91	1.02	1.13	1.08	1.17	1.06
R^2 3	0.86	1.11	0.80	1.03	1.09	1.08	0.99	1.05	0.99	0.99

σ_s^{old} : The within-subgroup true surrogate effects SD for main simulation. σ_s^{new} : Value for sensitivity analysis.
RR_{np}: Simulation root mean squared error from NP-RE that of PP-RE.
WR_{np}: Average 95% credible interval width from NP-RE over that of PP-RE.

Table 3: Partial and No-Pooling Models With Unequal Subgroup Sizes

Subgroup Size	Setup 2.1 (Truth)	PP-RE Summary	NP-RE Summary
9	α_1 (0)	0.01(-0.15,0.20)	0.00(-0.86,0.86)
12	α_2 (0)	0.01(-0.15,0.19)	0.01(-0.70,0.75)
24	α_3 (0)	-0.02(-0.17,0.14)	0.00(-0.27,0.35)
9	β_1 (-0.25)	-0.37(-0.85,0.11)	-0.26(-3.39,2.87)
12	β_2 (-0.35)	-0.41(-0.78,-0.06)	-0.39(-2.11,1.34)
24	β_3 (-0.6)	-0.57(-0.83,-0.36)	-0.60(-1.18,-0.17)
9	σ_{e1} (0.15)	0.10(0.02,0.26)	0.12(0.03,0.36)
12	σ_{e2} (0.115)	0.09(0.02,0.21)	0.10(0.03,0.27)
24	σ_{e3} (0.06)	0.08(0.02,0.18)	0.08(0.03,0.20)
9	R^2 1 (0.35)	0.63(0.07,0.98)	0.56(0.01,0.98)
12	R^2 2 (0.65)	0.71(0.16,0.98)	0.64(0.07,0.97)
24	R^2 3 (0.95)	0.88(0.51,1.00)	0.89(0.48,0.99)
Subgroup Size	Setup 2.2 (Truth)	PP-RE Summary	NP-RE Summary
9	α_1 (0)	-0.02(-0.18,0.15)	0.00(-0.60,0.68)
12	α_2 (0)	0.00(-0.16,0.17)	0.01(-0.67,0.72)
24	α_3 (0)	0.02(-0.14,0.19)	0.00(-0.44,0.44)
9	β_1 (-0.6)	-0.47(-0.90,-0.11)	-0.62(-2.86,1.32)
12	β_2 (-0.35)	-0.36(-0.71,-0.05)	-0.38(-2.00,1.13)
24	β_3 (-0.25)	-0.29(-0.55,-0.05)	-0.25(-0.96,0.45)
9	σ_{e1} (0.06)	0.11(0.01,0.26)	0.11(0.03,0.34)
12	σ_{e2} (0.115)	0.11(0.02,0.23)	0.11(0.03,0.28)
24	σ_{e3} (0.15)	0.11(0.04,0.21)	0.12(0.05,0.23)
9	R^2 1 (0.95)	0.73(0.15,0.99)	0.78(0.10,0.99)
12	R^2 2 (0.65)	0.63(0.11,0.97)	0.64(0.06,0.98)
24	R^2 3 (0.35)	0.52(0.09,0.90)	0.47(0.06,0.90)

Displayed are simulation average posterior medians and 95% credible intervals. The random-effects models were used for this demonstration.

Table 4: Full-Pooling and No-Pooling Models in All Simulation Setups with Bimodal True Surrogate Effects

Setup 1 (Truth)	FP-RE Median(95%CrI)	NP-RE Median(95%CrI)	FP-FE Median(95%CrI)	NP-FE Median(95%CrI)
α_1 (0)	0.00(-0.08,0.10)	0.01(-0.19,0.29)	-0.05(-0.12,0.03)	-0.05(-0.19,0.09)
α_2 (0)	0.00(-0.08,0.10)	0.01(-0.17,0.27)	-0.05(-0.12,0.03)	-0.05(-0.19,0.09)
α_3 (0)	0.00(-0.08,0.10)	0.01(-0.22,0.31)	-0.05(-0.12,0.03)	-0.05(-0.20,0.10)
β_1 (-0.45)	-0.46(-0.60,-0.33)	-0.47(-1.04,-0.08)	-0.35(-0.45,-0.26)	-0.34(-0.53,-0.17)
β_2 (-0.45)	-0.46(-0.60,-0.33)	-0.47(-0.96,-0.16)	-0.35(-0.45,-0.26)	-0.35(-0.54,-0.17)
β_3 (-0.45)	-0.46(-0.60,-0.33)	-0.46(-1.01,-0.05)	-0.35(-0.45,-0.26)	-0.35(-0.54,-0.17)
σ_{e1} (0.05)	0.07(0.03,0.14)	0.08(0.03,0.23)	0.08(0.03,0.15)	0.09(0.03,0.22)
σ_{e2} (0.05)	0.07(0.03,0.14)	0.08(0.03,0.22)	0.08(0.03,0.15)	0.09(0.03,0.23)
σ_{e3} (0.05)	0.07(0.03,0.14)	0.08(0.03,0.22)	0.08(0.03,0.15)	0.08(0.03,0.22)
R^2 1 (0.95)	0.91(0.64,0.99)	0.85(0.27,0.99)	0.91(0.68,0.99)	0.87(0.40,0.99)
R^2 2 (0.95)	0.91(0.64,0.99)	0.85(0.30,0.99)	0.91(0.68,0.99)	0.86(0.37,0.98)
R^2 3 (0.95)	0.91(0.64,0.99)	0.84(0.28,0.99)	0.91(0.68,0.99)	0.87(0.38,0.99)
Setup 2 (Truth)	FP-RE Median(95%CrI)	NP-RE Median(95%CrI)	FP-FE Median(95%CrI)	NP-FE Median(95%CrI)
α_1 (0)	0.00(-0.10,0.11)	-0.00(-0.32,0.30)	-0.05(-0.13,0.04)	-0.01(-0.14,0.12)
α_2 (0)	0.00(-0.10,0.11)	0.03(-0.43,0.52)	-0.05(-0.13,0.04)	-0.03(-0.17,0.12)
α_3 (0)	0.00(-0.10,0.11)	0.02(-0.49,0.74)	-0.05(-0.13,0.04)	-0.10(-0.29,0.09)
β_1 (-0.25)	-0.47(-0.67,-0.29)	-0.28(-1.35,0.88)	-0.34(-0.46,-0.23)	-0.21(-0.43,0.01)
β_2 (-0.35)	-0.47(-0.67,-0.29)	-0.42(-1.59,0.62)	-0.34(-0.46,-0.23)	-0.28(-0.49,-0.07)
β_3 (-0.6)	-0.47(-0.67,-0.29)	-0.62(-1.68,0.14)	-0.34(-0.46,-0.23)	-0.43(-0.66,-0.21)
σ_{e1} (0.15)	0.13(0.07,0.22)	0.12(0.04,0.28)	0.15(0.08,0.23)	0.12(0.04,0.28)
σ_{e2} (0.115)	0.13(0.07,0.22)	0.10(0.03,0.26)	0.15(0.08,0.23)	0.12(0.04,0.26)
σ_{e3} (0.06)	0.13(0.07,0.22)	0.09(0.03,0.25)	0.15(0.08,0.23)	0.10(0.03,0.26)
R^2 1 (0.35)	0.72(0.38,0.92)	0.49(0.04,0.93)	0.74(0.44,0.91)	0.58(0.08,0.93)
R^2 2 (0.65)	0.72(0.38,0.92)	0.65(0.08,0.97)	0.74(0.44,0.91)	0.72(0.15,0.96)
R^2 3 (0.95)	0.72(0.38,0.92)	0.85(0.29,0.99)	0.74(0.44,0.91)	0.87(0.40,0.99)
Setup 3 (Truth)	FP-RE Median(95%CrI)	NP-RE Median(95%CrI)	FP-FE Median(95%CrI)	NP-FE Median(95%CrI)
α_1 (0)	-0.00(-0.10,0.10)	-0.00(-0.28,0.27)	-0.06(-0.15,0.02)	-0.01(-0.14,0.12)
α_2 (0)	-0.00(-0.10,0.10)	0.02(-0.24,0.41)	-0.06(-0.15,0.02)	-0.07(-0.21,0.09)
α_3 (0)	-0.00(-0.10,0.10)	0.03(-0.31,0.59)	-0.06(-0.15,0.02)	-0.10(-0.29,0.09)
β_1 (-0.25)	-0.54(-0.73,-0.36)	-0.26(-1.22,0.74)	-0.39(-0.51,-0.27)	-0.21(-0.43,0.01)
β_2 (-0.6)	-0.54(-0.73,-0.36)	-0.66(-1.60,-0.10)	-0.39(-0.51,-0.27)	-0.43(-0.67,-0.21)
β_3 (-0.6)	-0.54(-0.73,-0.36)	-0.64(-1.51,-0.09)	-0.39(-0.51,-0.27)	-0.43(-0.66,-0.21)
σ_{e1} (0.15)	0.12(0.05,0.21)	0.12(0.04,0.28)	0.14(0.07,0.23)	0.12(0.04,0.28)
σ_{e2} (0.06)	0.12(0.05,0.21)	0.09(0.03,0.25)	0.14(0.07,0.23)	0.10(0.03,0.27)
σ_{e3} (0.06)	0.12(0.05,0.21)	0.09(0.03,0.25)	0.14(0.07,0.23)	0.10(0.03,0.26)
R^2 1 (0.35)	0.80(0.49,0.95)	0.48(0.03,0.93)	0.80(0.52,0.94)	0.58(0.08,0.93)
R^2 2 (0.95)	0.80(0.49,0.95)	0.88(0.33,0.99)	0.80(0.52,0.94)	0.86(0.38,0.98)
R^2 3 (0.95)	0.80(0.49,0.95)	0.87(0.31,0.99)	0.80(0.52,0.94)	0.87(0.40,0.99)
Setup 4 (Truth)	FP-RE Median(95%CrI)	NP-RE Median(95%CrI)	FP-FE Median(95%CrI)	NP-FE Median(95%CrI)
α_1 (0)	0.00(-0.10,0.12)	0.01(-0.29,0.29)	-0.04(-0.13,0.05)	-0.01(-0.14,0.12)
α_2 (0)	0.00(-0.10,0.12)	0.02(-0.38,0.44)	-0.04(-0.13,0.05)	-0.01(-0.16,0.14)
α_3 (0)	0.00(-0.10,0.12)	0.03(-0.31,0.56)	-0.04(-0.13,0.05)	-0.10(-0.29,0.09)
β_1 (-0.25)	-0.45(-0.65,-0.25)	-0.26(-1.25,0.79)	-0.33(-0.45,-0.20)	-0.21(-0.43,0.01)
β_2 (-0.25)	-0.45(-0.65,-0.25)	-0.28(-1.32,0.67)	-0.33(-0.45,-0.20)	-0.22(-0.45,0.01)
β_3 (-0.6)	-0.45(-0.65,-0.25)	-0.63(-1.47,-0.10)	-0.33(-0.45,-0.20)	-0.43(-0.66,-0.21)
σ_{e1} (0.15)	0.15(0.08,0.24)	0.12(0.04,0.28)	0.17(0.10,0.25)	0.12(0.04,0.28)
σ_{e2} (0.15)	0.15(0.08,0.24)	0.12(0.04,0.28)	0.17(0.10,0.25)	0.14(0.05,0.29)
σ_{e3} (0.06)	0.15(0.08,0.24)	0.09(0.03,0.25)	0.17(0.10,0.25)	0.10(0.03,0.26)
R^2 1 (0.35)	0.64(0.29,0.87)	0.48(0.04,0.93)	0.67(0.36,0.87)	0.58(0.08,0.93)
R^2 2 (0.35)	0.64(0.29,0.87)	0.50(0.03,0.94)	0.67(0.36,0.87)	0.56(0.06,0.92)
R^2 3 (0.95)	0.64(0.29,0.87)	0.87(0.31,0.99)	0.67(0.36,0.87)	0.87(0.40,0.99)

6 PP-RE vs PP-FE on CKD-EPI Trials (Application)

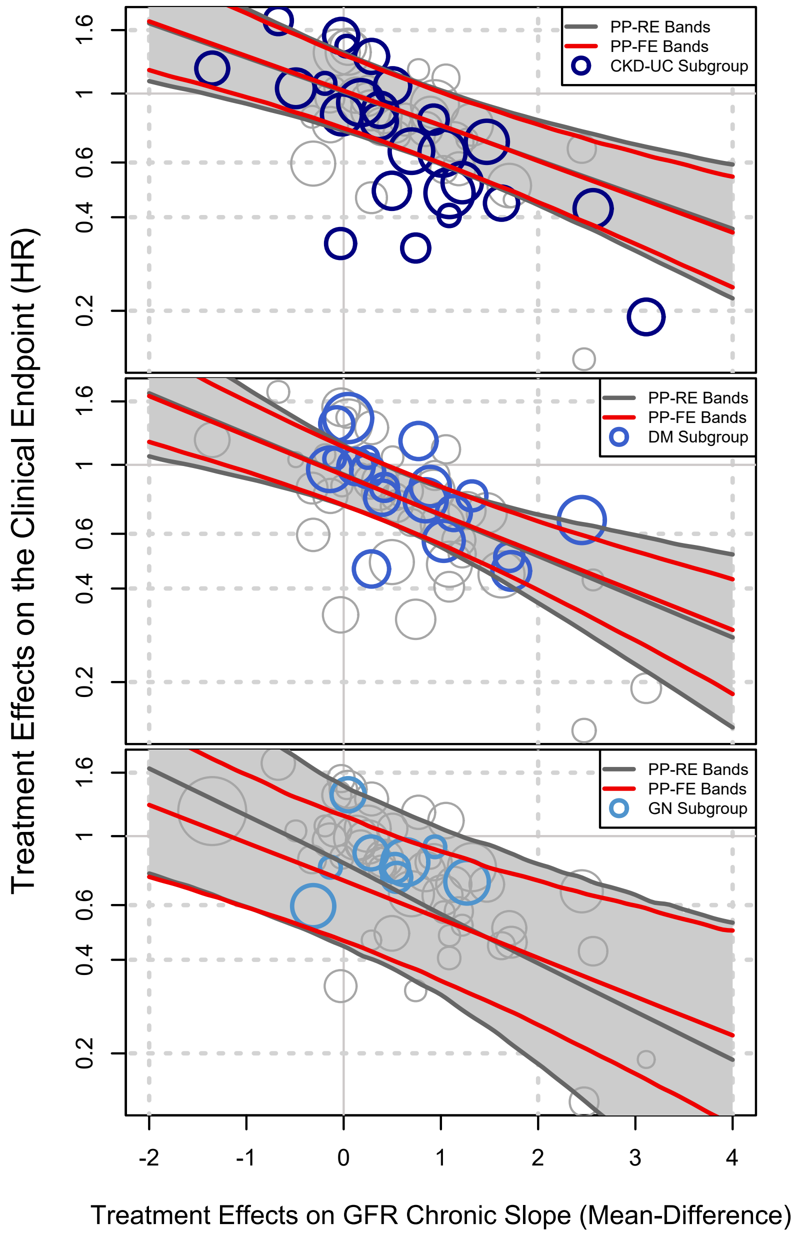

Figure 7: This is a trial-level scatter plot, displaying regression lines and prediction bands obtained from fitting the PP-RE and PP-FE models to the application analysis data with disease-defined subgroups (CKD-UC: chronic kidney disease; DM: diabetes; GN: glomerular diseases). The circles represent individual trials, with circle size proportional to the number of patients.

7 Additional Results from Application Analyses

Table 5: Meta-Regression Posteriors from Analyses With Disease-Defined Subgroups

Parameter Summarized	Diffuse Priors	Constrained Priors Set 1	Constrained Priors Set 2
Mean α	-0.07(-0.82,0.6)	-0.07(-0.54,0.32)	-0.06(-0.31,0.13)
Mean β	-0.3(-0.85,0.2)	-0.3(-0.68,0.02)	-0.3(-0.61,-0.06)
Mean σ_e	0.11(0.04,1.03)	0.1(0.04,0.65)	0.1(0.04,0.43)
Between-SG SD: α	0.23(0.02,1.84)	0.18(0.01,0.91)	0.11(0.01,0.36)
Between-SG SD: β	0.15(0.01,1.53)	0.13(0.01,0.77)	0.12(0.01,0.55)
Between-SG SD: $log(\sigma_e)$	0.88(0.05,4.1)	0.82(0.04,3.47)	0.64(0.04,2.44)
α_1 (CKD)	0.01(-0.11,0.14)	0.01(-0.11,0.14)	0.00(-0.11,0.13)
α_2 (Diabetes)	-0.07(-0.19,0.04)	-0.07(-0.19,0.04)	-0.07(-0.18,0.04)
α_3 (Glomerular Diseases)	-0.2(-0.57,0.07)	-0.18(-0.54,0.06)	-0.13(-0.45,0.06)
β_1 (CKD)	-0.25(-0.39,-0.13)	-0.25(-0.38,-0.13)	-0.25(-0.37,-0.13)
β_2 (Diabetes)	-0.30(-0.48,-0.13)	-0.30(-0.48,-0.14)	-0.30(-0.48,-0.15)
β_3 (Glomerular Diseases)	-0.35(-0.66,-0.16)	-0.35(-0.66,-0.17)	-0.35(-0.66,-0.18)
σ_{e1} (CKD)	0.11(0.02,0.23)	0.11(0.02,0.22)	0.11(0.03,0.22)
σ_{e2} (Diabetes)	0.07(0.01,0.18)	0.07(0.01,0.18)	0.08(0.01,0.18)
σ_{e3} (Glomerular Diseases)	0.11(0.01,0.70)	0.11(0.01,0.65)	0.10(0.01,0.59)

Constrained Priors Set 1 were based on the iterative procedure described in section 3.
Constrained Priors Set 2 are narrower than those of set 1, based on domain-specific reasoning.

Table 6: Meta-Regression Posteriors from Analyses With Intervention-Defined Subgroups

Parameter Summarized	Diffuse Priors	Constrained Priors Set 1	Constrained Priors Set 2
Mean α	0.02(-0.2,0.18)	0.02(-0.19,0.18)	0.02(-0.14,0.16)
Mean β	-0.41(-0.75,-0.13)	-0.41(-0.74,-0.13)	-0.4(-0.68,-0.18)
Mean σ_e	0.08(0.03,0.22)	0.07(0.03,0.21)	0.08(0.03,0.21)
Between-SG SD: α	0.11(0.01,0.43)	0.11(0.01,0.42)	0.08(0,0.27)
Between-SG SD: β	0.19(0.02,0.73)	0.18(0.02,0.7)	0.15(0.01,0.47)
Between-SG SD: $log(\sigma_e)$	0.66(0.03,2.49)	0.64(0.04,2.41)	0.57(0.03,2.02)
α_1 (Antiplatelet)	0.01(-0.21,0.2)	0.02(-0.21,0.2)	0.02(-0.18,0.18)
α_2 (DPP-4)	0.03(-0.14,0.19)	0.03(-0.13,0.19)	0.03(-0.12,0.18)
α_3 (Immunosuppressants)	-0.07(-0.48,0.15)	-0.06(-0.47,0.15)	-0.03(-0.39,0.14)
α_4 (Modify Blood Pressure))	0.06(-0.08,0.23)	0.06(-0.08,0.23)	0.05(-0.08,0.21)
α_5 (RASB vs CCB)	0(-0.31,0.24)	0(-0.31,0.23)	0.01(-0.24,0.2)
α_6 (RASB vs Control)	0.1(-0.06,0.29)	0.09(-0.06,0.29)	0.08(-0.06,0.25)
α_7 (SGLT-2)	0.01(-0.45,0.29)	0.01(-0.42,0.29)	0.02(-0.3,0.26)
β_1 (Antiplatelet)	-0.39(-1.03,0.34)	-0.39(-1.01,0.33)	-0.39(-0.88,0.14)
β_2 (DPP-4)	-0.4(-1.04,0.16)	-0.4(-1.01,0.14)	-0.39(-0.89,0.04)
β_3 (Immuosuppressants)	-0.47(-0.92,-0.23)	-0.47(-0.94,-0.24)	-0.46(-0.87,-0.24)
β_4 (Modify Blood Pressure)	-0.45(-0.84,-0.18)	-0.44(-0.83,-0.17)	-0.43(-0.78,-0.18)
β_5 (RASB vs CCB)	-0.41(-0.81,-0.06)	-0.41(-0.79,-0.06)	-0.41(-0.75,-0.14)
β_6 (RASB vs Control)	-0.5(-0.82,-0.25)	-0.49(-0.82,-0.24)	-0.47(-0.75,-0.23)
β_7 (SGLT-2)	-0.25(-0.49,0.01)	-0.25(-0.49,0.00)	-0.27(-0.48,-0.07)
σ_{e1} (Antiplatelet)	0.08(0.01,0.38)	0.08(0.01,0.36)	0.08(0.01,0.35)
σ_{e2} (DPP-4)	0.07(0.01,0.25)	0.07(0.01,0.24)	0.07(0.01,0.24)
σ_{e3} (Immunosuppressants)	0.08(0.01,0.56)	0.08(0.01,0.58)	0.09(0.01,0.56)
σ_{e4} (Modify Blood Pressure)	0.06(0.01,0.21)	0.07(0.01,0.21)	0.07(0.01,0.21)
σ_{e5} (RASB vs CCB)	0.07(0.01,0.25)	0.07(0.01,0.24)	0.07(0.01,0.24)
σ_{e6} (RASB vs Control)	0.07(0.01,0.19)	0.07(0.01,0.19)	0.07(0.01,0.19)
σ_{e7} (SGLT-2)	0.09(0.01,0.41)	0.09(0.01,0.4)	0.1(0.02,0.39)

Constrained Priors Set 1 were based on the iterative procedure described in manuscript Section 3. Constrained Priors Set 2 are narrower than those of set 1, based on domain-specific reasoning.

Table 7: Prediction for CVD Studies Left-Out of Model Fitting

	FP-RE Model	PP-RE (Diffuse)	PP-RE (Constrained Set 1)	PP-RE (Constrained Set 2)
NSG Study 1	0.86(0.59,1.25)	0.8(0.04,15.67)	0.81(0.16,4.09)	0.82(0.35,1.82)
NSG Study 2	0.80(0.42,1.53)	0.76(0.03,15.27)	0.77(0.13,4.28)	0.77(0.26,2.12)
NSG Study 3	0.86(0.59,1.24)	0.80(0.05,17.85)	0.81(0.15,3.67)	0.81(0.35,1.86)
NSG Study 4	1.08(0.67,1.80)	0.98(0.06,14.64)	0.98(0.20,4.58)	0.99(0.43,2.19)
NSG Study 5	0.96(0.57,1.62)	0.89(0.04,15.70)	0.88(0.17,4.59)	0.89(0.35,2.22)
NSG Study 6	1.21(0.72,2.15)	1.05(0.06,18.63)	1.06(0.20,5.17)	1.07(0.45,2.47)
NSG Study 7	0.96(0.62,1.48)	0.87(0.05,16.48)	0.88(0.19,4.26)	0.88(0.38,1.99)

Constrained Priors Set 1 were based on the iterative procedure described in manuscript Section 3.
Constrained Priors Set 2 are narrower than those of set 1, based on domain-specific reasoning.
NSG: "New Subgroup"

Table 8: Prediction for Studies of Intervention-Defined Subgroups Left-Out of Model Fitting

	FP-RE Model	PP-RE (Diffuse)	PP-RE (Constrained Set 1)	PP-RE (Constrained Set 2)
NSG Study 1	0.87(0.55,1.37)	0.83(0.34,1.96)	0.83(0.35,1.83)	0.84(0.43,1.60)
NSG Study 2	0.96(0.52,1.77)	0.92(0.30,2.51)	0.93(0.32,2.36)	0.93(0.38,2.12)
NSG Study 3	0.79(0.39,1.59)	0.73(0.25,2.17)	0.74(0.26,2.04)	0.74(0.30,1.81)
NSG Study 4	0.81(0.55,1.18)	0.77(0.31,1.77)	0.77(0.33,1.65)	0.78(0.40,1.42)
NSG Study 5	0.72(0.47,1.12)	0.72(0.23,1.87)	0.72(0.25,1.78)	0.73(0.31,1.52)
NSG Study 6	0.90(0.62,1.31)	0.85(0.38,1.74)	0.86(0.40,1.70)	0.86(0.48,1.51)
NSG Study 7	1.08(0.69,1.73)	1.06(0.51,2.13)	1.06(0.52,2.07)	1.06(0.59,1.86)
NSG Study 8	0.85(0.55,1.33)	0.82(0.32,1.93)	0.82(0.33,1.79)	0.83(0.40,1.55)
NSG Study 9	1.02(0.69,1.53)	0.99(0.40,2.19)	0.99(0.42,2.09)	0.99(0.51,1.80)
NSG Study 10	0.65(0.45,0.90)	0.60(0.19,1.73)	0.60(0.20,1.62)	0.61(0.26,1.29)
NSG Study 11	0.98(0.65,1.49)	0.94(0.42,1.94)	0.94(0.44,1.86)	0.94(0.50,1.67)
NSG Study 12	1.11(0.25,5.10)	1.08(0.19,6.23)	1.09(0.20,5.91)	1.10(0.21,5.60)
NSG Study 13	0.86(0.59,1.26)	0.82(0.35,1.77)	0.82(0.36,1.74)	0.83(0.44,1.50)
NSG Study 14	0.89(0.62,1.27)	0.85(0.39,1.75)	0.85(0.40,1.65)	0.86(0.48,1.47)
NSG Study 15	0.98(0.72,1.34)	0.94(0.46,1.81)	0.94(0.47,1.71)	0.95(0.57,1.52)

Constrained Priors Set 1 were based on the iterative procedure described in manuscript Section 3.
Constrained Priors Set 2 are narrower than those of set 1, based on domain-specific reasoning.
NSG: "New Subgroup"

Table 9: Prediction for Disease Subgroups Used in Model Fitting (Part 1)

		FP-RE	PP-RE P1	PP-RE P2	PP-RE P3
Study Name	Subgroup	Predicted HR	Predicted HR	Predicted HR	Predicted HR
ESG Study 6	Diabetes	1(0.45,2.2)	1.06(0.48,2.33)	1.06(0.48,2.33)	1.06(0.48,2.31)
ESG Study 7	Diabetes	1.07(0.48,2.35)	1.13(0.52,2.50)	1.13(0.51,2.49)	1.12(0.51,2.46)
ESG Study 8	Diabetes	0.89(0.57,1.39)	0.94(0.62,1.44)	0.94(0.61,1.43)	0.93(0.61,1.42)
ESG Study 9	Diabetes	0.98(0.63,1.55)	1.04(0.68,1.60)	1.04(0.67,1.59)	1.03(0.67,1.57)
ESG Study 10	Diabetes	0.89(0.64,1.25)	0.95(0.70,1.30)	0.95(0.70,1.29)	0.94(0.69,1.28)
ESG Study 11	Diabetes	0.69(0.4,1.18)	0.75(0.44,1.26)	0.75(0.44,1.26)	0.75(0.44,1.24)
ESG Study 12	Diabetes	0.89(0.62,1.28)	0.95(0.68,1.32)	0.95(0.68,1.32)	0.94(0.67,1.30)
ESG Study 13	Diabetes	0.98(0.58,1.65)	1.04(0.62,1.72)	1.04(0.63,1.72)	1.03(0.62,1.71)
ESG Study 14	Diabetes	0.49(0.32,0.72)	0.55(0.37,0.79)	0.55(0.37,0.78)	0.55(0.37,0.78)
ESG Study 15	Diabetes	0.66(0.39,1.12)	0.71(0.42,1.18)	0.71(0.43,1.17)	0.71(0.43,1.17)
ESG Study 1	Diabetes	0.6(0.38,0.93)	0.66(0.43,0.99)	0.66(0.43,0.99)	0.66(0.43,0.99)
ESG Study 16	Diabetes	1(0.66,1.51)	1.05(0.71,1.57)	1.05(0.71,1.56)	1.05(0.70,1.54)
ESG Study 17	Diabetes	0.67(0.47,0.92)	0.73(0.53,0.97)	0.72(0.53,0.97)	0.72(0.52,0.97)
ESG Study 18	Diabetes	0.73(0.47,1.12)	0.79(0.52,1.18)	0.79(0.52,1.18)	0.78(0.52,1.17)
ESG Study 19	Diabetes	0.73(0.5,1.05)	0.79(0.56,1.10)	0.79(0.56,1.09)	0.79(0.56,1.09)
ESG Study 20	Diabetes	0.76(0.52,1.1)	0.82(0.58,1.15)	0.82(0.58,1.15)	0.82(0.58,1.13)
ESG Study 21	Diabetes	0.85(0.58,1.26)	0.91(0.63,1.31)	0.91(0.63,1.30)	0.90(0.63,1.30)
ESG Study 22	Diabetes	0.75(0.51,1.08)	0.81(0.57,1.13)	0.81(0.57,1.13)	0.80(0.57,1.12)
ESG Study 23	Diabetes	0.75(0.53,1.04)	0.80(0.59,1.09)	0.80(0.59,1.08)	0.80(0.59,1.07)
ESG Study 24	Diabetes	0.79(0.53,1.17)	0.85(0.58,1.22)	0.85(0.58,1.21)	0.85(0.58,1.21)
ESG Study 2	Diabetes	0.97(0.55,1.68)	1.03(0.60,1.76)	1.02(0.60,1.75)	1.01(0.59,1.73)
ESG Study 25	CKD	0.89(0.61,1.29)	0.86(0.63,1.15)	0.86(0.63,1.15)	0.86(0.63,1.15)
ESG Study 26	CKD	0.7(0.45,1.06)	0.67(0.46,0.95)	0.67(0.46,0.95)	0.67(0.46,0.96)
ESG Study 27	CKD	0.86(0.58,1.27)	0.83(0.59,1.13)	0.83(0.59,1.13)	0.83(0.59,1.14)
ESG Study 28	CKD	0.92(0.41,2.07)	0.88(0.41,1.93)	0.88(0.40,1.92)	0.89(0.41,1.94)
ESG Study 29	CKD	0.9(0.5,1.64)	0.86(0.49,1.47)	0.86(0.49,1.47)	0.86(0.49,1.49)
ESG Study 30	CKD	1.08(0.24,4.8)	1.07(0.25,4.77)	1.08(0.25,4.75)	1.08(0.25,4.69)
ESG Study 3	CKD	0.64(0.44,0.91)	0.60(0.44,0.80)	0.60(0.44,0.79)	0.60(0.44,0.80)
ESG Study 31	CKD	0.77(0.38,1.58)	0.73(0.37,1.40)	0.72(0.37,1.41)	0.72(0.37,1.43)
ESG Study 32	CKD	0.99(0.45,2.17)	0.98(0.46,2.06)	0.97(0.46,2.07)	0.97(0.46,2.10)
ESG Study 33	CKD	0.87(0.4,1.89)	0.84(0.40,1.77)	0.84(0.40,1.77)	0.84(0.40,1.76)
ESG Study 34	CKD	0.93(0.6,1.43)	0.90(0.62,1.30)	0.90(0.62,1.30)	0.90(0.62,1.30)
ESG Study 35	CKD	0.72(0.4,1.27)	0.69(0.40,1.16)	0.68(0.40,1.16)	0.68(0.40,1.17)
ESG Study 36	CKD	0.71(0.47,1.06)	0.66(0.46,0.92)	0.66(0.47,0.93)	0.66(0.47,0.92)
ESG Study 37	CKD	0.75(0.37,1.54)	0.72(0.36,1.43)	0.72(0.36,1.42)	0.72(0.36,1.42)
ESG Study 38	CKD	0.81(0.38,1.7)	0.76(0.37,1.56)	0.77(0.37,1.56)	0.77(0.37,1.56)
ESG Study 39	CKD	0.83(0.56,1.23)	0.79(0.57,1.09)	0.79(0.57,1.09)	0.79(0.57,1.10)
ESG Study 40	CKD	0.67(0.42,1.06)	0.64(0.42,0.95)	0.64(0.42,0.95)	0.64(0.42,0.95)
ESG Study 41	CKD	0.82(0.52,1.29)	0.79(0.53,1.17)	0.79(0.53,1.16)	0.79(0.53,1.18)
ESG Study 42	CKD	0.87(0.59,1.27)	0.83(0.60,1.14)	0.83(0.60,1.13)	0.83(0.60,1.14)
ESG Study 43	CKD	0.96(0.65,1.41)	0.92(0.66,1.26)	0.92(0.67,1.26)	0.92(0.67,1.26)
ESG Study 44	CKD	0.58(0.36,0.93)	0.54(0.35,0.82)	0.54(0.35,0.81)	0.54(0.35,0.82)
ESG Study 45	CKD	0.7(0.42,1.16)	0.68(0.43,1.07)	0.68(0.43,1.07)	0.68(0.43,1.07)
ESG Study 46	CKD	0.92(0.54,1.58)	0.88(0.55,1.41)	0.88(0.55,1.41)	0.88(0.55,1.41)

P1: Diffuse; P2: Constrained set 1; P2: Constrained set 2. Summary includes PPD median, 2.5^{th} and 97.5^{th} percentiles. ESG: "Existing Subgroup"

Table 10: Prediction for Disease Subgroups Used in Model Fitting (Part 2)

		FP-RE	PP-RE P1	PP-RE P2	PP-RE P3
Study	Subgroup	Predicted HR	Predicted HR	Predicted HR	Predicted HR
ESG Study 47	CKD	0.84(0.52,1.34)	0.80(0.53,1.21)	0.80(0.53,1.21)	0.80(0.52,1.21)
ESG Study 48	CKD	0.91(0.67,1.25)	0.88(0.70,1.09)	0.89(0.70,1.09)	0.88(0.70,1.09)
ESG Study 49	CKD	0.82(0.34,1.99)	0.80(0.34,1.87)	0.80(0.34,1.85)	0.80(0.34,1.86)
ESG Study 50	CKD	1.42(0.46,4.41)	1.48(0.49,4.39)	1.47(0.49,4.37)	1.49(0.49,4.36)
ESG Study 4	CKD	0.89(0.4,2.03)	0.85(0.39,1.85)	0.85(0.39,1.85)	0.85(0.39,1.85)
ESG Study 51	Glomerular	0.8(0.33,1.98)	0.64(0.16,2.45)	0.66(0.17,2.49)	0.68(0.18,2.49)
ESG Study 52	Glomerular	0.68(0.18,2.55)	0.45(0.09,2.16)	0.46(0.09,2.15)	0.48(0.10,2.20)
ESG Study 53	Glomerular	1.03(0.51,2.11)	0.86(0.34,2.18)	0.88(0.36,2.17)	0.92(0.40,2.20)
ESG Study 54	Glomerular	0.64(0.32,1.26)	0.41(0.08,1.50)	0.41(0.08,1.47)	0.44(0.09,1.49)
ESG Study 55	Glomerular	0.5(0.2,1.27)	0.34(0.10,1.08)	0.34(0.11,1.07)	0.36(0.12,1.07)
ESG Study 56	Glomerular	1.47(0.72,2.99)	1.31(0.51,3.32)	1.33(0.55,3.26)	1.38(0.60,3.27)
ESG Study 57	Glomerular	2.18(0.65,7.56)	2.52(0.58,11.31)	2.52(0.6,11.32)	2.62(0.67,11.26)
ESG Study 58	Glomerular	0.48(0.18,1.31)	0.29(0.08,1.00)	0.29(0.08,1.01)	0.31(0.09,1.01)
ESG Study 59	Glomerular	0.75(0.33,1.67)	0.56(0.2,1.56)	0.57(0.21,1.54)	0.60(0.22,1.55)
ESG Study 5	Glomerular	0.89(0.28,2.83)	0.72(0.2,2.62)	0.73(0.21,2.61)	0.77(0.22,2.62)

P1: Diffuse; P2: Constrained set 1; P2: Constrained set 2. Summary includes PPD median, 2.5^{th} and 97.5^{th} percentiles. ESG: "Existing Subgroup"

Table 11: Prediction for Intervention Subgroups Used in Model Fitting (Part 1)

		FP-RE	PP-RE P1	PP-RE P2	PP-RE P3
Study	Subgroup	Predicted HR	Predicted HR	Predicted HR	Predicted HR
ESG Study 6	Antiplatelet	1.08(0.7,1.7)	1.07(0.66,1.74)	1.07(0.66,1.74)	1.08(0.67,1.72)
ESG Study 7	Antiplatelet	1(0.63,1.61)	1.00(0.59,1.69)	1.00(0.59,1.69)	1.00(0.60,1.67)
ESG Study 8	Antiplatelet	1.03(0.6,1.81)	1.05(0.56,1.93)	1.04(0.57,1.92)	1.05(0.58,1.89)
ESG Study 1	DPP-4 Inhibitor	0.94(0.66,1.34)	0.96(0.68,1.35)	0.96(0.67,1.35)	0.96(0.68,1.34)
ESG Study 9	DPP-4 Inhibitor	1.06(0.64,1.76)	1.07(0.65,1.77)	1.07(0.65,1.75)	1.07(0.65,1.75)
ESG Study 10	DPP-4 Inhibitor	1.07(0.72,1.61)	1.08(0.73,1.61)	1.08(0.73,1.60)	1.08(0.73,1.60)
ESG Study 11	Immunosuppression	0.83(0.32,2.14)	0.71(0.16,2.90)	0.71(0.16,2.88)	0.73(0.18,2.89)
ESG Study 12	Immunosuppression	0.69(0.18,2.6)	0.48(0.09,2.41)	0.49(0.09,2.39)	0.50(0.10,2.40)
ESG Study 13	Immunosuppression	1.08(0.53,2.18)	0.94(0.37,2.44)	0.95(0.38,2.44)	0.99(0.40,2.43)
ESG Study 14	Immunosuppression	0.64(0.3,1.36)	0.46(0.08,1.73)	0.47(0.09,1.75)	0.48(0.10,1.75)
ESG Study 15	Immunosuppression	1.63(0.81,3.32)	1.62(0.69,3.99)	1.62(0.69,3.96)	1.67(0.73,4.01)
ESG Study 2	Immunosuppression	2.36(0.68,8.32)	2.94(0.68,14.32)	2.9(0.66,14.42)	2.93(0.69,13.87)
ESG Study 16	Immunosuppression	0.45(0.16,1.23)	0.26(0.07,0.91)	0.26(0.07,0.93)	0.28(0.07,0.95)
ESG Study 17	Immunosuppression	0.76(0.33,1.7)	0.59(0.20,1.59)	0.59(0.20,1.65)	0.61(0.21,1.66)
ESG Study 18	Immunosuppression	0.92(0.29,2.86)	0.77(0.21,2.76)	0.77(0.22,2.75)	0.81(0.23,2.84)
ESG Study 19	Low vs Usual BP	0.93(0.65,1.33)	0.94(0.67,1.31)	0.94(0.67,1.31)	0.93(0.67,1.30)
ESG Study 20	Low vs Usual BP	1.05(0.48,2.27)	1.06(0.49,2.25)	1.06(0.49,2.28)	1.05(0.49,2.27)
ESG Study 21	Low vs Usual BP	0.91(0.43,1.98)	0.90(0.42,1.90)	0.90(0.42,1.91)	0.89(0.42,1.90)
ESG Study 22	Low vs Usual BP	0.67(0.43,1.04)	0.65(0.39,1.02)	0.65(0.39,1.01)	0.65(0.40,1.02)
ESG Study 23	Low vs Usual BP	0.9(0.62,1.32)	0.92(0.62,1.33)	0.91(0.62,1.33)	0.91(0.63,1.32)
ESG Study 24	Low vs Usual BP	0.97(0.54,1.77)	1.03(0.58,1.91)	1.03(0.58,1.90)	1.03(0.57,1.87)
ESG Study 25	Low vs Usual BP	1.2(0.76,1.93)	1.31(0.82,2.13)	1.31(0.82,2.12)	1.29(0.81,2.08)
ESG Study 26	RASB vs CCB	0.7(0.46,1.06)	0.64(0.41,0.98)	0.64(0.41,0.98)	0.65(0.42,0.99)
ESG Study 27	RASB vs CCB	1.14(0.52,2.49)	1.11(0.50,2.42)	1.11(0.50,2.44)	1.13(0.51,2.44)
ESG Study 28	RASB vs CCB	0.74(0.51,1.05)	0.68(0.46,0.98)	0.68(0.46,0.98)	0.69(0.46,0.98)
ESG Study 29	RASB vs CCB	0.96(0.41,2.27)	0.83(0.36,2.03)	0.83(0.36,2.03)	0.85(0.36,2.06)

P1: Diffuse; P2: Constrained set 1; P2: Constrained set 2. Summary includes PPD median, 2.5^{th} and 97.5^{th} percentiles.

Table 12: Prediction for Intervention Subgroups Used in Model Fitting (Part 2)

		FP-RE	PP-RE P1	PP-RE P2	PP-RE P3
Study	Subgroup	Predicted HR	Predicted HR	Predicted HR	Predicted HR
ESG Study 30	RASB vs CONTROL	0.9(0.61,1.31)	0.90(0.64,1.25)	0.90(0.64,1.26)	0.89(0.64,1.25)
ESG Study 31	RASB vs CONTROL	0.93(0.6,1.43)	0.96(0.64,1.41)	0.96(0.64,1.40)	0.95(0.64,1.40)
ESG Study 32	RASB vs CONTROL	0.95(0.69,1.3)	0.97(0.74,1.27)	0.97(0.74,1.25)	0.96(0.73,1.24)
ESG Study 3	RASB vs CONTROL	0.97(0.43,2.18)	0.96(0.42,2.18)	0.96(0.43,2.15)	0.95(0.42,2.13)
ESG Study 33	RASB vs CONTROL	0.83(0.43,1.62)	0.82(0.43,1.57)	0.82(0.42,1.57)	0.82(0.43,1.56)
ESG Study 34	RASB vs CONTROL	0.66(0.38,1.13)	0.66(0.38,1.12)	0.66(0.38,1.12)	0.66(0.38,1.11)
ESG Study 35	RASB vs CONTROL	1.06(0.49,2.32)	1.14(0.54,2.43)	1.13(0.54,2.39)	1.13(0.53,2.39)
ESG Study 36	RASB vs CONTROL	0.99(0.65,1.5)	1.03(0.70,1.49)	1.02(0.70,1.49)	1.01(0.70,1.47)
ESG Study 37	RASB vs CONTROL	0.73(0.41,1.29)	0.71(0.40,1.25)	0.71(0.40,1.25)	0.71(0.40,1.25)
ESG Study 38	RASB vs CONTROL	0.72(0.48,1.05)	0.66(0.47,0.92)	0.66(0.47,0.93)	0.66(0.47,0.94)
ESG Study 39	RASB vs CONTROL	0.78(0.54,1.11)	0.76(0.53,1.05)	0.76(0.53,1.05)	0.76(0.53,1.05)
ESG Study 40	RASB vs CONTROL	0.48(0.19,1.21)	0.47(0.18,1.19)	0.47(0.18,1.20)	0.47(0.18,1.20)
ESG Study 41	RASB vs CONTROL	0.77(0.38,1.57)	0.75(0.37,1.56)	0.75(0.37,1.54)	0.75(0.37,1.52)
ESG Study 42	RASB vs CONTROL	0.83(0.39,1.74)	0.82(0.39,1.69)	0.81(0.39,1.69)	0.81(0.39,1.68)
ESG Study 43	RASB vs CONTROL	0.57(0.36,0.9)	0.51(0.32,0.79)	0.51(0.32,0.80)	0.52(0.33,0.81)
ESG Study 44	RASB vs CONTROL	0.76(0.52,1.1)	0.75(0.51,1.06)	0.75(0.51,1.06)	0.75(0.52,1.06)
ESG Study 45	RASB vs CONTROL	0.88(0.62,1.26)	0.88(0.64,1.19)	0.88(0.64,1.18)	0.87(0.64,1.19)
ESG Study 46	RASB vs CONTROL	0.71(0.42,1.19)	0.70(0.42,1.15)	0.70(0.42,1.15)	0.70(0.42,1.15)
ESG Study 4	RASB vs CONTROL	0.76(0.54,1.04)	0.73(0.53,0.96)	0.72(0.53,0.96)	0.73(0.54,0.97)
ESG Study 47	RASB vs CONTROL	0.86(0.35,2.07)	0.84(0.35,2.07)	0.84(0.35,2.05)	0.85(0.35,2.03)
ESG Study 48	RASB vs CONTROL	1.5(0.49,4.55)	1.75(0.60,5.04)	1.74(0.59,5.08)	1.70(0.58,4.98)
ESG Study 49	SGLT-2 Inhibitor	0.69(0.4,1.15)	0.74(0.40,1.28)	0.74(0.41,1.29)	0.74(0.41,1.28)
ESG Study 5	SGLT-2 Inhibitor	0.45(0.3,0.65)	0.55(0.30,0.81)	0.55(0.31,0.80)	0.54(0.30,0.79)
ESG Study 50	SGLT-2 Inhibitor	0.62(0.44,0.87)	0.69(0.44,0.99)	0.69(0.43,0.98)	0.69(0.44,0.98)
ESG Study 51	SGLT-2 Inhibitor	0.57(0.37,0.87)	0.65(0.38,1.00)	0.65(0.39,1.00)	0.65(0.38,1.00)

P1: Diffuse; P2: Constrained set 1; P2: Constrained set 2. Summary includes PPD median, 2.5^{th} and 97.5^{th} percentiles.

8 Study Acronyms for CKD-EPI Studies

Table 13: Study Acronyms

Acronym	Complete Study Name
AASK	African American Study of Kidney Disease and Hypertension
ABCD	Appropriate Blood Pressure Control in Diabetes trial
ADVANCE	Action in Diabetes and Vascular Disease: Preterax and Diamicron MR Controlled Evaluation trial
AIPRI	The Angiotensin-converting-enzyme Inhibition on Progressive Renal Insufficiency trial
ALTITUDE	Aliskiren Trial in Type 2 Diabetes Using Cardiorenal Endpoints
CanPREVENT	Canadian Prevention of Renal and Cardiovascular Endpoints Trial
CSG	Collaborative Study Group
EMPA-REG OUTCOME	Empagliflozin Cardiovascular Outcome Event Trial in Type 2 Diabetes Mellitus Patients
HALT-PKD	Halt Progression of Polycystic Kidney Disease study
HKVIN	Hong Kong study using Valsartan in IgA Nephropathy
IDNT	Irbesartan Diabetic Nephropathy Trial
MASTERPLAN	Multifactorial Approach and Superior Treatment Efficacy in Renal Patients with the Aid of Nurse Practitioners study
MDRD Study	Modification of Diet in Renal Disease study
ORIENT	Olmesartan Reducing Incidence of Endstage Renal Disease in Diabetic Nephropathy Trial
REIN	Ramipril Efficacy In Nephropathy study
RENAAL	Reduction of Endpoints in NIDDM with the Angiotensin II Antagonist Losartan study
ROAD	Renoprotection of Optimal Antiproteinuric Doses study
SHARP	Study of Heart and Renal Protection
STOP-IgAN	Supportive Versus Immunosuppressive Therapy for the Treatment of Progressive IgA Nephropathy trial
SUN-MACRO	Sulodexide Macroalbuminuria trial
